# Slowdown in China's methane emission growth

**DOI:** 10.1093/nsr/nwae223

**Published:** 2024-06-26

**Authors:** Min Zhao, Xiangjun Tian, Yilong Wang, Xuhui Wang, Philippe Ciais, Zhe Jin, Hongqin Zhang, Tao Wang, Jinzhi Ding, Shilong Piao

**Affiliations:** State Key Laboratory of Tibetan Plateau Earth System, Resources and Environment (TPESRE), Institute of Tibetan Plateau Research, Chinese Academy of Sciences, Beijing 100101, China; State Key Laboratory of Tibetan Plateau Earth System, Resources and Environment (TPESRE), Institute of Tibetan Plateau Research, Chinese Academy of Sciences, Beijing 100101, China; University of Chinese Academy of Sciences, Beijing 101408, China; State Key Laboratory of Tibetan Plateau Earth System, Resources and Environment (TPESRE), Institute of Tibetan Plateau Research, Chinese Academy of Sciences, Beijing 100101, China; Sino-French Institute for Earth System Science, College of Urban and Environmental Sciences, Peking University, Beijing 100871, China; Laboratoire des Sciences du Climat et de l'Environnement, LSCE/IPSL, CEA-CNRS-UVSQ, Université Paris-Saclay, Gif-sur-Yvette 91191, France; State Key Laboratory of Tibetan Plateau Earth System, Resources and Environment (TPESRE), Institute of Tibetan Plateau Research, Chinese Academy of Sciences, Beijing 100101, China; Institute of Carbon Neutrality, College of Urban and Environmental Sciences, Peking University, Beijing 100871, China; Institute of Atmospheric Physics, Chinese Academy of Sciences, Beijing 100029, China; State Key Laboratory of Tibetan Plateau Earth System, Resources and Environment (TPESRE), Institute of Tibetan Plateau Research, Chinese Academy of Sciences, Beijing 100101, China; State Key Laboratory of Tibetan Plateau Earth System, Resources and Environment (TPESRE), Institute of Tibetan Plateau Research, Chinese Academy of Sciences, Beijing 100101, China; State Key Laboratory of Tibetan Plateau Earth System, Resources and Environment (TPESRE), Institute of Tibetan Plateau Research, Chinese Academy of Sciences, Beijing 100101, China; Institute of Carbon Neutrality, College of Urban and Environmental Sciences, Peking University, Beijing 100871, China

**Keywords:** methane emissions, greenhouse gases, data assimilation, China, Global ObservatioN-based system for monitoring Greenhouse GAses for methane

## Abstract

The unprecedented surge in global methane levels has raised global concerns in recent years, casting a spotlight on China as a pivotal emitter. China has taken several actions to curb the methane emissions, but their effects remain unclear. Here, we developed the Global ObservatioN-based system for monitoring Greenhouse GAses for methane (GONGGA-CH_4_) and assimilate GOSAT XCH_4_ observations to assess changes in China's methane emissions. We find the average rate of increase in China's methane emissions (0.1 ± 0.3 Tg CH_4_ yr^−2^) during 2016–2021 slowed down compared to the preceding years (2011–2015) (0.9 ± 0.5 Tg CH_4_ yr^−2^), in contrast to the concurrent acceleration of global methane emissions. As a result, the contribution of China to global methane emissions dropped significantly. Notably, the slowdown of China's methane emission is mainly attributable to a reduction in biogenic emissions from wetlands and agriculture, associated with the drying trend in South China and the transition from double-season to single-season rice cropping, while fossil fuel emissions are still increasing. Our results suggest that GONGGA-CH_4_ provides the opportunity for independent assessment of China's methane emissions from an atmospheric perspective, providing insights into the implementation of methane-related policies that align with its ambitious climate objectives.

## Teaser

China's methane emission growth has slowed significantly due to reduced emissions from agriculture and wetlands, with the Global ObservatioN-based system for monitoring Greenhouse GAses for methane (GONGGA-CH_4_) system providing crucial insights into these changes.

## INTRODUCTION

Atmospheric methane concentrations have become three times larger than preindustrial levels [1]. As a result, the effective radiative forcing of methane amounts to 1.19 (0.81–1.58) W m^−2^, contributing about one-third of the current global warming attributed to anthropogenic emissions of greenhouse gases [2], which is next only to carbon dioxide (CO_2_). The rapid growth of atmospheric methane is a result of escalating methane emissions linked to industrialization and intensified crop and livestock production [3–5]. Given methane's shorter atmospheric lifetime (∼10 years) compared to CO_2_, the mitigation of methane emissions holds promise as a prompt and impactful strategy for achieving the imperative objective of restraining global warming to below the 1.5°C threshold [2]. This goal is pivotal for aligning human society with the climate objectives delineated in the Paris Agreement [6].

China, currently the world's largest emitter of anthropogenic methane [7], has prioritized reducing methane emissions in the ‘14th Five-Year Plan’, and has also made a declaration to develop a comprehensive National Action Plan on methane to curb its emission in the 2020s [8]. Coal mining and rice cultivation are the two largest sources of methane emissions in China, followed by waste treatment and livestock [9]. Accurately quantifying methane emissions, as well as its sectorial contributions, is crucial for tracking emission changes and assessing the effectiveness of mitigation efforts, which is recommended in the Intergovernmental Panel on Climate Control Sixth Assessment Report for National Greenhouse Gas Inventories to be based on atmospheric inversion. In addition, the unprecedented surge in global methane growth rates in 2020 and 2021 marked a record-breaking increase of 15.2 ± 0.4 and 17.6 ± 0.5 parts per billion per year (ppb yr^−1^) [10], arousing further interest in exploring regional contributions to the large year-to-year change in global methane budget.

Large uncertainties remain in quantifying national methane budgets and its spatio-temporal change [11–18]. For example, the ensemble of 22 atmospheric inversions in the Global Methane Budget report from the Global Carbon Project (GCP) presents a nearly two-fold difference in estimates for China's average methane emissions between 2011 and 2017 (43−68 Tg CH_4_ yr^−1^) and substantial variations in emission trends (−0.1 to 1.5 Tg CH_4_ yr^−2^) [19], with the greatest uncertainty lying in anthropogenic emissions [20]. Some inversion analyses of observations from satellite and surface networks have shown that annual methane emissions in China increased by ∼1 Tg CH_4_ yr^−2^ from 2000 to 2010 [14,17], primarily due to coal mining. But whether such a large trend was sustained after 2010 was unclear. For instance, Miller *et al.* [15] conducted atmospheric inversions based on Greenhouse gases Observing SATellite (GOSAT) observations and argued that China's methane emissions were still increasing rapidly (1.1 ± 0.4 Tg CH_4_ yr^−2^) from 2010 to 2015, with coal emissions being the dominant driver, while bottom-up inventories reported that coal mining emissions in China peaked around 2012 and have since either decreased or stabilized [12,21]. Zhang *et al.* [18] showed with an atmospheric inversion that China's energy policy prioritizing the phase-out of small coal mines results in contrasting trends in methane emissions from coal mining across different regions, yet with an overall increase in coal mine emissions from 2010 to 2016, and that China's agricultural and environmental policies (e.g. promoting straw return), mainly aiming to enhance crop productivity and air quality, may have also led to enhanced methane emissions from rice cultivation [18]. In 2016, China issued the Work Plan for Controlling Greenhouse Gas Emissions During the 13th Five-Year Plan Period [22], with a focus on controlling agriculture greenhouse gas emissions. But the change of methane emissions after this government work plan and the Paris Agreement has not been assessed yet.

Here, we developed a new atmospheric inversion system, called the Global ObservatioN-based system for monitoring Greenhouse GAses for methane (GONGGA-CH_4_), to assimilate measurements of vertically integrated columns of dry air mole fractions of methane (XCH_4_) from the GOSAT UoL retrievals (see Methods section) and estimated global methane fluxes during 2011–2021, at a spatial resolution of 2° × 2.5° (latitude × longitude). The GONGGA-CH_4_ system uses GEOS-Chem to simulate the atmospheric transport and chemical oxidation of methane in the atmosphere, and adopts the novel dual-pass inversion strategy of the GONGGA carbon inversion system (Fig. S1), which can distinguish the model-data mismatch caused by biases due to atmospheric transport, chemical oxidization and methane fluxes [23]. The GONGGA-CH_4_ uses the nonlinear least-squares four-dimensional variational data assimilation (NLS-4DVar) algorithm to accurately solve the non-linear inverse problem [24–27]. Three distinct prior information sources [7,28–30] are utilized to in the inversion analysis of China's annual methane emission trends (see Supplementary Table).

## RESULTS

### Methane emissions in China during the last decade

**Figure 1.**
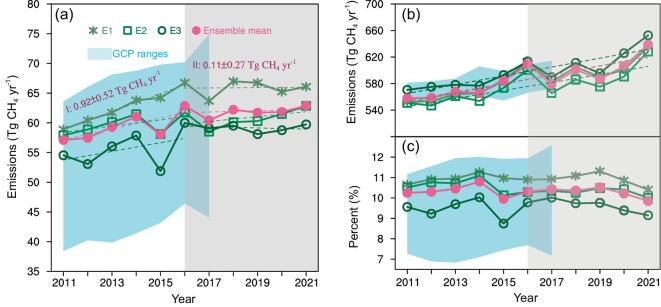
China's annual methane emissions and their global contribution. (a) China's methane emissions from 2011 to 2021 and the trend in 2011–2016 (Phase I with white background) and 2016–2021 (Phase II with grey background). Stars, squares, circles, and dots represent the different results with various prior emissions and ensemble mean. Light-blue shaded area represents the range of Global Carbon Project (GCP) inversions; (b) global methane emissions from 2011 to 2021 and the trend in 2011–2021; (c) proportion of China’s methane emissions to the global total from 2011 to 2021.

As Fig. 1a shows, GONGGA-CH_4_ estimates that China's annual mean total methane emission during 2011–2021 is 60.4 ± 3.6 Tg CH_4_ yr^−1^ (the uncertainty denotes the standard deviation of three inversions using different priors), whose contemporary estimates (59.5 ± 3.5 Tg CH_4_ yr^−1^ during 2011–2017) is consistent with the estimates of GOSAT-based inversions from the GCP global methane budget report (45.4–70.0 Tg CH_4_ yr^−1^) [19], as well as other studies using GOSAT XCH_4_ measurements over similar study periods (50.0–68.0 Tg CH_4_ yr^−1^) [15,16,18] (Fig. 1a and Fig. S2). At the same time, the global methane emission is estimated to be 586.8 ± 11.3 Tg CH_4_ yr^−1^ and was rapidly rising from 2011 to 2021, with the largest increase in 2021.

China's methane emissions increased by ∼10% (5.8 Tg CH_4_ yr^−1^) over the past decade, but the increase is not constant (Fig 1a). Specifically, methane emissions increased from 2011 to 2016 with a mean rate of 0.9 ± 0.5 Tg CH_4_ yr^−2^ (*p* < 0.01), despite a small emission in 2015 likely due to the strong El Niño event. The increasing trend before 2016 is consistent with the previous top-down estimates from GCP over similar time periods (1.1 ± 0.5 Tg CH_4_ yr^−2^). Then the methane emission trend slows down after 2016 to 0.1 ± 0.3 Tg CH_4_ yr^−2^ (*p* > 0.5) (Fig. 1a, Fig. S3). To test the robustness of the inversion results and the interpretation of trends, we employ three sets of distinct prior methane emission products (see Methods section). All three inversions consistently show an increase of methane emissions before 2016 and a slowdown afterwards, while the prior emissions show divergent emission changes in both periods before and after 2016 (Fig. S4). Notably, global methane emissions increased from 609.0 ± 7.5 Tg CH_4_ yr^−1^ in 2016 to 638.8 ± 12.6 Tg CH_4_ yr^−1^ in 2021 (Fig. 1b). As a result, China's share of methane emissions in the global total has declined from 10.3% in 2016 to 9.8% in 2021, and this signal is consistently observed across all three inversions (Fig. 1c).

**Figure 2.**
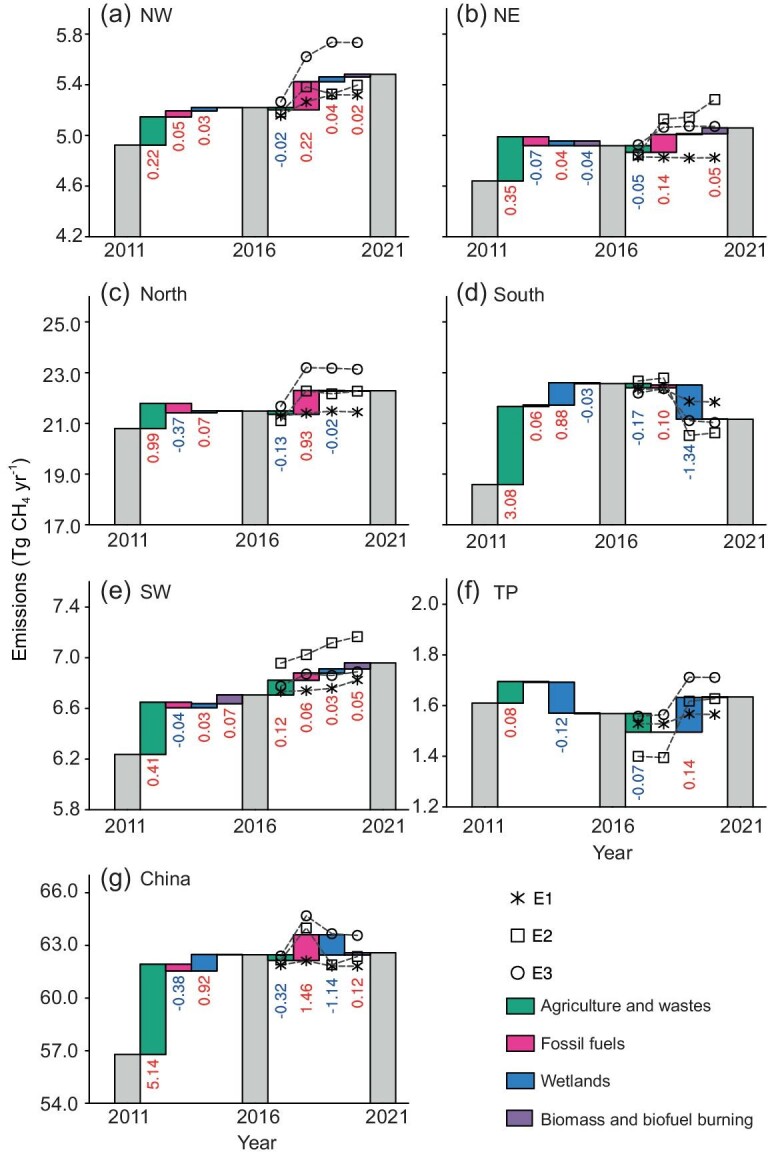
Changes in China's methane emissions for different regions and sectors. The methane emission changes over (a) Northwest (NW), (b) Northeast (NE), (c) North, (d) South, (e) Southwest (SW), (f) Tibetan Plateau (TP), and (g) China. Green, red, blue, and purple bars represent Agriculture and waste, Fossil fuels, Wetlands, and Biomass and Biofuel Burning. Each graph represents the accumulative changes in different sectors of methane emissions for 2011–2016 and 2016–2021. The asterisk, circle, and square represent the results of the three experiments.

Spatially, methane emissions have been persistently increasing over most regions of China (Fig. 2) during the last decade, except South China and the Tibetan Plateau. The regions used in this study are shown in Fig. S5. Emissions in South China largely increased before 2016, but decreased after 2016, in pace with the national total emissions. The Tibetan Plateau, on the contrary, shows opposite emission changes with a decreasing trend before 2016 and an increasing trend afterwards. In GONGGA-CH_4_, we simultaneously optimize the emissions of different sectors by dedicatedly designing the control vector and leveraging distinct spatio-temporal patterns of emissions from different sectors among the ensemble samples (see Methods section). In the following section, we will discuss the key sectors driving these emission changes with a focus on the post-2016 period. Moreover, we analyze the data representing the underlying processes of emissions from different sectors to elucidate their drivers.

### Reduction in wetland emission

**Figure 3.**
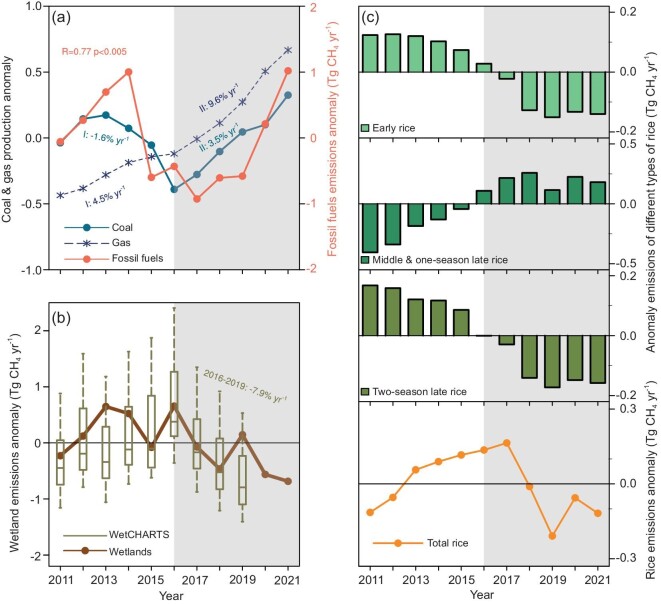
Drivers influencing changes in China's methane emissions. (a) Fossil fuels sector: annual anomaly in coal production (10^9^ tons yr^−1^) and gas production (10^11^ m^3^ yr^−1^) from 2011–2021 from National Statistics Bureau, along with fossil fuel methane emissions (Tg CH_4_ yr^−1^) in GONGGA-CH_4_. (b) Wetland sector: box plot depicting annual anomaly of wetland emissions (Tg CH_4_ yr^−1^) in WetCHARTs dataset (2011–2019) and line chart depicting wetland emissions (Tg CH_4_ yr^−1^) of GONGGA-CH_4_ (2011–2021) over South China. (c) Rice cultivation sector: annual anomaly of early rice, middle and one-season late rice, two-season late rice, and total rice methane emissions (Tg CH_4_ yr^−1^) for the period 2011–2021, based on the Bottom-Up method (in SI text).

China's wetland emissions increased by 0.9 ± 0.6 Tg CH_4_ yr^−1^ from 2011 to 2016, and decreased by 1.1 ± 0.9 Tg CH_4_ yr^−1^ from 2016 to 2021. The reduction of wetland emissions after 2016 mainly occurred in South China, while the emissions over the Tibetan Plateau slightly increased after 2016. Wetland emissions in other regions only have marginal changes. Our results are consistent with the WetCHARTs v1.3.1 dataset, encompassing nine terrestrial biosphere models, all of which indicate a decrease in wetland methane emissions over South China (Fig. 3b, Fig. S6) and an increase over the Tibetan Plateau.

Higher water content can create anaerobic conditions, and is favored for methane production [31] over warm low-latitude regions [32]. In South China, we found that the climate becomes drier after 2016, marked by a reduction in liquid water equivalent (LWE) height anomalies observed by the Gravity Recovery and Climate Experiment Follow-On (GRACE-FO) satellite, which is a proxy for water content in wetland systems [33,34] (Fig. S7). As a result, wetland methane emissions closely follow such a decreasing trend of LWE (r = 0.40, *p* = 0.2; Fig. S7). Over the Tibetan Plateau, LWE generally increased after 2016, but its correlation with wetland emissions was less pronounced (Fig. S7), as snow accumulation and low surface temperature can suppress methanogens’ activity [31,32,35,36] and disturbs methane production.

### Small decrease in agriculture and waste emissions

In the agriculture and waste sector, the emissions increased rapidly by 5.1 ± 3.8 Tg CH_4_ yr^−1^ from 2011 to 2016, but slightly decreased by 0.32 ± 0.25 Tg CH_4_ yr^−1^ from 2016 to 2021. Spatially, agriculture and waste methane emissions from all the regions increased remarkably before 2016, but levelled off or slightly decreased afterwards except in southwest China (Fig. 2d and Fig. S8). Within this sector, national landfill production increased steadily by 24% and 22% before and after 2016, which cannot explain the change of emission growth (Fig. S9) [37]. Without significant changes in waste management practices, waste-induced methane emissions are mainly driven by the level of urbanization, which increased by 7% and 5.8% during the two periods, respectively (Fig. S9), and can potentially contribute to the slowdown of emission growth but is far from sufficient to result in the decrease of emissions after 2016. In the agriculture sector, the livestock population, which determines the methane emissions from enteric fermentation and manure management, shows opposite trends than the total emissions of agriculture and waste, with a decrease before 2016 and an increase afterwards (Fig. S9).

Given the increasing trends in the above-mentioned emission processes, we hypothesized that the emission reduction since 2016 should be related to changing methane emissions from rice cultivation. We found that the area of early-season (−10.5%) and double-season (−7.6%) late rice decreased since 2016, while the area of mid-season and late-season rice slightly increased, resulting in an overall decrease in rice cultivation area by 2.7% (Fig. S10) [37]. We conducted a bottom-up estimate of methane emissions from rice cultivation using the reported emission factors [38] and national statistics of rice cultivation area, accounting for different types of rice and different cultivation practices (Supplementary Information Text). The calculated methane emission from rice cultivation in 2021 is smaller than that in 2016 by 0.25 ± 0.11 Tg CH_4_ yr^−1^ (Fig. 3c), which explains more than 75% of the total methane emission change in the agriculture and waste sector. Spatially, this ‘double-to-single rice’ transition mainly took place in South China, where two-season rice cultivation is dominant (Fig. S11) [37]. This explains why South China has the largest drop of emissions from the agriculture and waste sector.

We also explored how the increased straw return, which was implemented to increase crop yield and improve air quality, affected methane emissions in the past decade. A former study has found this practice contributed to the rapid increase of agricultural methane emissions before 2016 [18]. We find that a larger fraction of straw return could enhance methane emissions from cropland (Fig. S12) and China's straw return fractions are steadily increasing from 18.52% in the 2000s to 46% in recent years [39,40], which is unlikely to contribute to the decrease of methane emissions.

### Increase in fossil fuel emission

Although overall methane emission has slowed down since 2016, the fossil fuel sector shows a remarkable increasing trend of 1.5 ± 1.1 Tg CH_4_ yr^−1^ after 2016 (Fig. 2g). As coal is China's main energy source and supports 44% of power generation and 47% of industry (including both energy and non-energy use) [37], recent changes in China's fossil fuel methane emissions are mostly determined by changes in coal production. For example, between 2011 and 2016, China's fossil fuel methane emissions first increased before 2013 but then decreased from 2013 to 2016, which follows coal production (Fig. 3a). The regional fluctuations in coal production before 2016 are consistent with China's energy policy to consolidate large coal mines and to close local small coal mines. But as small coal mines were closed, coal production rose again after 2016. As a result, fossil fuel methane emissions synchronized with the coal production and increased steadily after 2016, especially in North China (0.9 ± 0.7 Tg CH_4_ yr^−1^) and Northwest China (0.2 ± 0.1 Tg CH_4_ yr^−1^) (Figs S13 and S14) [37], where ∼80% of the nation's coal production took place (Fig. S15) [37,41].

In the meantime, emissions from abandoned small coal mines following the coal mine consolidation policy may have also contributed to the increase in coal methane emissions after 2016 [42] as abandoned mines can release a considerable amount of methane at 40%–90% of the initial emission rate in the first 3–4 years after closure [43]. Apart from the coal production, China's natural gas production has continued to increase, with doubled growth rate after 2016 (Fig. 3a). Particularly in northwest China, there has been a 133.8% surge from 2016 to 2021 (Fig. S14d) [37]. China's oil production, also a source of methane emission, has declined since its peak in 2015 (Fig. S16) [37]. Currently, the emissions from natural gas and oil in China account for less than 10% of total methane emissions from fossil fuels, so their contribution to the change of fossil fuel emissions might be smaller compared to coal.

## DISCUSSION

Our inversion indicates that China's methane emission growth has slowed down after 2016, compared to the preceding period. The deceleration of China's methane emissions since 2016 can be primarily attributed to a combination of reduced wetland emissions, and a slight decrease in agriculture and waste emissions, which offset the increase in fossil fuel emissions.

In recent years, the global methane growth rates have reached record highs, with 2020 and 2021 registering the highest levels. The deceleration of China's methane emissions has resulted in a decrease in China's contribution to the global total. This reduction is primarily attributed to decreasing emissions in the wetland and agricultural sectors, while global wetland emissions have risen in recent years, particularly in 2020 and 2021. Northern tropical and boreal regions were identified as major contributors to the global increase in wetland emissions [34]. Our study reveals that inland water emissions from South China are decreasing due to a drying environment, underscoring the intricate impacts of atmospheric circulation on regional climates.

In addition, human activities also play a crucial role in shaping wetlands and their emissions. In 2022, China enacted a new law aimed at protecting wetlands, with the goal of placing 55% of its wetland areas under national protection [44]. Consequently, it is anticipated that a substantial portion of wetland will be rehabilitated and expanded in the forthcoming years. The side-effect of such actions will present a significant challenge in controlling methane emissions from this natural source in the near future.

Our result shows that the transition of rice cultivation is probably responsible for the stabilization or reduction of China's agricultural methane emissions since 2016. However, it is reported that in some places, the decline in the double-season rice planting area has halted [37] (Fig. 3) due to government incentives and the adoption of high-yielding varieties. This calls for innovative approaches to control methane emissions from agricultural activities. For example, the Methane Emission Control Action Plan [45] issued in November 2023 promotes actions like resource utilization of livestock and poultry waste, strengthening water management in paddy fields with water-saving irrigation techniques, collecting methane from sewage treatment, classified recycling for waste disposal, etc. Field experiments also revealed that slag and biochar application [46], high-stalk rice cultivation [47], landfill gas collection and flaring [48] can also reduce methane emissions from the agriculture and waster sector. The effectiveness of such actions on controlling methane emissions will thus need a continuous and timely assessment.

Despite the overall slowdown of China's methane emissions, the substantial increase in energy demand and thus fossil fuel methane emissions since 2016, following a temporary reduction of emissions due to the policy to close small coal mines, raises a cautionary warning. This suggests that controlling methane emissions remains quite challenging in China, with the energy sector being a focal point for future mitigation efforts. This is especially crucial considering that the reductions in emissions from wetlands and agriculture may not be sustained in the future, as discussed above.

It should be acknowledged that GOSAT XCH_4_, a pioneering mission for measuring atmospheric methane levels from space, samples the atmosphere with a surface footprint of about 10 km, and these footprints are separated by more than 200 km. Such a sampling approach creates significant gaps in the spatial coverage, which brings great challenges in inferring methane fluxes at regional to sub-national scales. As a result, the inverted fluxes can be sensitive to the prior ones [49]. However, despite the large spread of prior fluxes and their trends among our three experiments, they consistently show a slowdown of China's methane emissions after 2016. In addition, the substantial increase in the emission from rice cultivation and a reduction in fossil fuel emission before 2016 found in this study were also reported by a recent study [18] which assimilated both satellite and *in-situ* measurements, corroborating our findings on the emission changes.

Despite the overall consistency in long-term emission changes, notable discrepancies in spatial distribution between our study and that of Zhang *et al.* [18] emerge when zooming in to the provincial level (Fig. S17), which may be addressed with dense coverage from newly launched satellites like TROPOMI and MethaneSAT and the ones being prepared like GOSAT-GW and CO2M.

Apart from the limitations in observations, the uncertainty of atmospheric inversion is also influenced by atmospheric transport model, configuration of inversion systems, and choice of prior fluxes. For example, biases in the modelling of vertical mixing and large-scale circulation were found to significantly impact the inverted fluxes at national to continental scales [50,51]. In this study, we assessed the impacts of the hyperparameters of the GONGGA-CH_4_ system, and found that they had marginal influences on the inverted methane emissions of China (Fig. S18). The wide range of estimates across different inversion systems in the Global Methane Budget report encompasses various sources of uncertainties, including the optimization algorithm, varying atmospheric transport, and prior assumptions. While detailed inversion techniques, improved modelling of transport and better prior fluxes may improve inversion results, it remains difficult to definitively conclude that one inversion model is superior to the other. Therefore, model intercomparison and the use of independent constraints are crucial for evaluating model performances.

The GONGGA-CH_4_ system is built with the capability to assimilate atmospheric observations, once they are available, and track global and regional methane budgets. Currently, the timeliness for the inversion depends on the availability of input data, with the satellite observations and meteorological data being the dominant limiting factor. In this study, we revisit China's methane emissions and extend the time period beyond the study of Zhang *et al.* [18]. We found that the emission trends have quickly shifted beyond 2016, highlighting the critical need for continuous monitoring of methane emissions. In the future, we hope to regularly update our inversion flux product at a similar frequency with the release of observations, helping us to track methane emissions and contributing to policy formulation.

## METHODS

### GEOS-Chem model for methane

We utilize the GEOS-Chem chemical transport model (CTM) (version 14.0.0) with a 2° × 2.5° horizontal resolution and 47 vertical layers to characterize the relationship between surface methane emissions and atmospheric methane concentrations. The model is forced by the meteorological data of Modern-Era Retrospective Analysis for Research and Applications, version 2 (MERRA-2), which is provided by the National Aeronautics and Space Administration (NASA). Spatially resolved methane emission estimates from various products, covering anthropogenic and natural sources, were collected to form three sets of priors (Table S1). The 3D monthly OH and Cl atom concentration fields provided by the default GEOS-Chem configuration were used to calculate the main methane removal through tropospheric OH oxidation, as well as other minor loss processes such as stratospheric OH oxidation and the tropospheric oxidation of Cl atoms. Such a climatology OH field without inter-annual variability is used to simulate the atmospheric oxidation of CH_4_ as some studies have suggested that global OH levels have remained relatively constant over the past several decades [52] and can be buffered against short-term anthropogenic and natural perturbations [53]. In addition, our dual-pass inversion strategy is less sensitive to varying OH fields than traditional inversions because any bias induced by OH oxidation, as well as by the atmospheric transport, is corrected in the first inversion channel of each inversion window as the initial methane field (see GONGGA-CH_4_ inversion system).

The initial fields are taken from 1 February 2010 methane concentration data provided on the GEO-Chem website, which we spin-up for 11 months using two-pass assimilation, so that the analysis data begins on 1 January 2011 and ends on 31 December 2021.

### GOSAT XCH_4_ data

The TANSO-FTS instrument on the GOSAT satellite measures column-averaged dry air methane mixing ratios in the shortwave infrared (1.65 μm) through solar backscatter with near-unit sensitivity [54,55]. The satellite, positioned in a polar sun-synchronous orbit, captured a circular pixel with a 10 km diameter at around 13:00 local time. The GOSAT spectra have maintained consistent data quality without significant drift or degradation since recording began. We employed the University of Leicester version 9.0 CO_2_ proxy method (https://data.ceda.ac.uk/neodc/gosat/data/ch4/nceov1.0/CH4_GOS_OCPR/, last accessed: 9 May 2023) [56]. The data has been extensively validated against ground column observations from the Total Carbon Column Observing Network (TCCON) [57]. High-quality retrievals with “xch4_quality_flag = 0”, spanning from 1 January 2011 to 31 December 2021), were assimilated in GONGGA-CH4 to optimize methane fluxes. The assimilated retrievals amount to 5 116 811 in total.

### GONGGA-CH_4_ inversion system

The GONGGA-CH_4_ atmospheric inversion system uses the NLS-4DVar algorithm to top-down optimize global surface methane emission fluxes. The optimized methane fluxes within each assimilation window are calculated by the following equation:


(1) \begin{eqnarray*}
{{F}_{tot}}\!\left( {i,j,t,\lambda } \right) &=& {{\lambda }_{{wetland}}}\left( {i,j} \right)\! \times \! {{F}_{{wetland}}}\left( {i,j,t} \right)\\
&& +\, {{\lambda }_{{fossil}}}\left( {i,j} \right) \times {{F}_{{fossil}}}\left( {i,j,t} \right)\\
&& +\, {{\lambda }_{{agriculture}/waste}}\left( {i,j} \right)\\
&&\quad \times {{F}_{{agriculture}/waste}}\left( {i,j,t} \right)\\
&& +\, {{\lambda }_{{fire}}}\left( {i,j} \right) \times {{F}_{{fire}}}\left( {i,j,t} \right)\\
&& +\, {{F}_{{others}}}\left( {i,j,t} \right),
\end{eqnarray*}

where $\lambda = {{( {{{\lambda }_{{wetland}}},{{\lambda }_{{fossil}}},{{\lambda }_{{agriculture}/waste}},}}} {{{{{\lambda }_{{fire}}}} )}^{\mathrm{T}}}$ is a set of linear scale factor vectors, and ${{F}_e}(e\! =\! \textit{wetland},\textit{fossil},\textit{agriculture}/waste,\textit{fire},\textit{others})$ with prior methane fluxes from three inventories, where *wetland* refers to both natural and managed wetland but excluding other inland water (e.g. lakes and rivers), *fire* refers to biomass burning and biofuel burning, and *other* includes all other emissions including non-wetland inland water, land geological sources, termites and oceanic sources. The GONGGA-CH_4_ system uses the observed methane concentrations (here is GOSAT XCH_4_) to optimize the $\lambda $ by the NLS-4DVar algorithm, and the optimized *posteriori* fluxes are obtained by multiplying the prior fluxes with the optimized $\lambda $. In the above equation, *i* and *j* denote grid points and *t* denotes time.

In GONGGA-CH_4_, we solve for the separate four main sectors. Such a categorization considers the origin of different methane sources, and follows the reporting convention of the Global Methane Budget report [19]. Specifically, wetland emission is mainly of natural origin, while emissions from agriculture and waste, fossil fuel, and fire are mainly of anthropogenic origin. Among the anthropogenic emissions, agriculture emissions are generated through biogenic processes, while fossil fuel and fire emissions are characterized by distinct combustion processes. Such an approach that separately solves for individual sectors has been adopted by some previous inversion systems [29,58]. It is built on the fact that while the individual sectors may overlap in some grid cells and time windows, they are distinctly different at large scales and longer time periods as optimized variables. For example, wetlands and rice cultivation may overlap in grid cells over South China, but their distributions over the Tibetan Plateau differ significantly. In addition, the cropping areas of rice may have tiny emissions in the non-growing seasons, while wetlands may have a different seasonal cycle with substantial emissions in warm winter. These distinctions are captured through ensemble sampling and the Random State Variables [59] approach (detailed below), ensuring the adequacy of its spatio-temporal patterns and facilitating the separation of emissions from different sectors. Moreover, we use Tikhonov regularization to optimally adjust the propagation of the prior uncertainty, and then combine it with the NLS-4DVar to achieve the sector optimization separately [60].

The initial samples ${{\lambda }_l}$($l = 1, \cdot \cdot \cdot ,36$) of the first assimilation window is built by the following equation:


(2) \begin{eqnarray*}
{{\lambda }_l}\left( {i,j} \right) = \frac{{\sum\nolimits_{k = 0}^S {{{F}_{a,l}}\left( {i,j,{{t}_k}} \right)} }}{{\sum\nolimits_{k = 0}^S {F\left( {i,j,{{t}_k}} \right)} }},
\end{eqnarray*}

where ${{t}_S} - {{t}_0}$(=14 days) is the length of the assimilation window, $F( {i,j,{{t}_k}} )$ is the prior flux for the initial assimilation window, ${{F}_{a,l}}( {i,j,{{t}_k}} )$ is the prior flux for the corresponding sequence window for different months (12 months per year, 36 months in total) over three consecutive years (covering the year in which the initial window is located). Using the Random State Variables method [59], 36 samples are initially transformed into 18 samples, and the negative signs are added to these 18 samples, resulting in a total of 36 samples (${{\lambda }_l}$, $l = 1, \cdot \cdot \cdot ,36$). Setting ${{{{\bf P}}}_x} = ( {\lambda _1^{\prime}, \cdot \cdot \cdot ,\lambda _N^{\prime}} )$, and $\lambda _l^{\prime} = \lambda _l^{} - \lambda _b^{}$, $\lambda _b^{} = {{( {1, \cdot \cdot \cdot ,1} )}^{\mathrm{T}}}$ is for the first window. In turn, the atmospheric CTM ${{M}_{{{t}_k} \to {{t}_{k + 1}}}}( { \cdot , \cdot } )$ is used for ensemble simulations and background simulations:


(3) \begin{eqnarray*}
{{c}_{k + 1,l}} = {{M}_{{{t}_k} \to {{t}_{k + 1}}}}\left( {{{c}_{k,l}},{{F}_{tot}}\left( {i,j,t,{{\lambda }_l}} \right)} \right)
\end{eqnarray*}

and


(4) \begin{eqnarray*}
{{c}_{k + 1,b}} = {{M}_{{{t}_k} \to {{t}_{k + 1}}}}\left( {{{c}_{k,b}},{{F}_{tot}}\left( {i,j,t,{{\lambda }_b}} \right)} \right),
\end{eqnarray*}

where ${{c}_k}$ is the simulated three-dimensional profile concentration of methane at time ${{t}_k}$, ${{c}_{0,b}} = {{c}_b}$, ${{c}_{0,l}} = {{c}_b}$ and ${{c}_b}$ is the background methane concentration at ${{t}_0}$.

Next, using the averaging kernel of [61], the simulated methane concentration profile is integrated in the observation operator to calculate the methane simulated column concentration as follows:


(5) \begin{eqnarray*}
{\mathrm{XCH}}_4^m = {\mathrm{XCH}}_4^a + {{{{\bf h}}}^T}{{\bf A}}\left( {c - {{c}_a}} \right),
\end{eqnarray*}

where ${\mathrm{XCH}}_4^m$ is the methane column concentration for the model simulation, i.e. the simulated observation obtained, ${\mathrm{XCH}}_4^a$ is the prior column concentration provided by the GOSAT data, ${{\bf h}}$ is the barometric weight function, ${{\bf A}}$ is the average kernel matrix, *c* is the methane profile for the model simulation, and ${{c}_a}$ is the prior methane profile provided by GOSAT. For convenience, ${\mathrm{XCH}}_4^m$ is denoted later by *y*. ${{y}_l}$ and ${{y}_b}$ are obtained by entering ${{c}_{k,l}}$ and ${{c}_{k,b}}$ into Eq. (5) and marking ${{{{\bf P}}}_y} = ( {y_1^{\prime}, \cdot \cdot \cdot ,y_N^{\prime}} )$, and $y_l^{\prime} = y_l^{} - y_b^{}$. Alternatively, assuming that the observation vector is $y_{obs,k}^{}$ (and its observation error covariance matrix is ${{{{\bf R}}}_k}$), and $y_{obs,k}^{\prime} = y_{obs,k}^{} - y_{k,b}^{}$.

According to the NLS-4DVar assimilation method described by [26], the surface emission flux of methane was optimized at each assimilation window by solving for the minimal value of the following cost function:


(6) \begin{eqnarray*}
J(x) &=& 1/2{{(x - {{x}_a})}^T}{{{{\bf B}}}^{ - 1}}(x - {{x}_a})\\
&& +\, 1/2\sum\limits_{k = 0}^S {{{{[{{y}_k} - {{H}_k}{{M}_{t \to {{t}_k}}}(x)]}}^T}{{{{\bf R}}}^{ - 1}}}\\
&&\quad {\times [{{y}_k} - {{H}_k}{{M}_{t \to {{t}_k}}}(x)]} ,
\end{eqnarray*}

where *x* is the state variable, *x_a_* is the prior estimate, **B** is the prior error covariance matrix. In GONGGA-CH_4_, the prior error covariance matrix **B** can be approximated by the ensemble covariance matrix **Be**: ${{\bf B}} \approx {{\bf Be}} = \frac{{{{{{\bf P}}}_x}\ {{\bf P}}_x^{\mathrm{T}}}}{{{\mathrm{N - 1}}}}$, where **P***_x_* is the ensemble of state vector perturbations. For the first inversion window, it is constructed from the ensemble samples. It continuously evolves with sample updates after each inversion window [23]. To avoid the sampling error problem and pseudo-correlation between fluxes at remote locations, an ensemble extension localization scheme is used on **B** [62], and the localization radius is 1000 km. *y_k_* is the methane observation, $H( \cdot )$is the observation operator, and the subscript *k* represents the moment, *y_k_* denotes the observation at the moment *t_k_*, with a total of S + 1 observations. ${{M}_{t \to {{t}_k}}}$is the CTM of simulated state vector changes, and **R** is the observation error covariance matrix and composed of errors inherent in the observation data and any other uncertainties that are not controlled by the inversion (e.g. transportation error and representation error).

The derivation of the NLS-4DVar formula and the localization techniques employed in solving for the minimal value of the cost function refers to [26] and [62]. In summary, Eq. (6) can be converted into a least squares Eq. (7) after a series of mathematical transformations, and a Gauss–Newton iterative scheme is used to solve the non-linear least squares problem to yield the optimal state variable x*:


(7) \begin{eqnarray*}
x_{}^* = x_a^{} + {{\bf P}}_x^{}\beta ,
\end{eqnarray*}

which can be expressed as a linear combination of prior values *x_a_* plus state perturbations. *β* is the vector of weighted coefficients of the perturbation, **P***_x_* is the ensemble of state vector perturbations. After several (∼3) iterations, the final optimal solution x* is obtained and combined with the GEOS-Chem model, Eq. (1) and Eq. (3), to generate the methane *a posteriori* flux and the assimilated methane concentration.

The ensemble samples are updated from the second window onwards and the sample update formula is


(8) \begin{eqnarray*}
{{\bf P}}_{x,w + 1}^{} = {{{{\bf P}}}_{x,w}}{{{{\bf V}}}_2}{{\Phi }^T},
\end{eqnarray*}

where ${{\bf P}}_{x,w + 1}^{}$ denotes the set of perturbed samples for the $w + 1$ window and ${{{{\bf P}}}_{x,w}}$ denotes the set of perturbed samples for the *w* window, $\Phi $ is a random orthogonal matrix. The matrix ${{{{\bf V}}}_2}$ is related to the observation error covariance matrix and ${{{{\bf P}}}_y}$, with specific reference to [26]. The method has the advantage of being well able to maintain sample dispersion. As the optimized fluxes are all sources of methane emissions, negative x* values need to be allowed to be positive and randomly multiplied by a small number to ensure that their physical properties are accurate.

The GONGGA-CH_4_ system employs the dual-pass inversion strategy in GONGGA to differentiate model simulation biases caused by errors in initial methane concentration from those due to surface methane fluxes [23]. As shown in Fig. S1, this strategy starts with a methane concentration channel inversion, where the optimization variable is the initial methane concentration with a window of 7 days. It is assumed that prior flux errors are marginal for this short duration, and the model simulation bias is mainly due to errors in the initial methane concentration related to uncertainty in atmospheric transport and chemical oxidation in the previous window. This ensures that biases in atmospheric transport and chemical oxidation will not accumulate during the inversion. The independent evaluation of modeled atmospheric methane driven by posterior fluxes against observations from TCCON sites and ObsPack surface stations shows that the biases at all stations across the globe and in different regions including East Asia are close to zero during the whole inversion period (Supplementary Information Text and Figs S19–S22). Furthermore, the results from a sensitivity test driven by a varying OH fields (Supplementary Information Text and Fig. S23) consistently show a slowdown of China's methane emissions after 2016, verifying the effectiveness of this methane concentration channel.

The second channel is the methane flux channel, where the optimization variable is the surface methane flux with a window of 14 days. Assuming that the errors in the initial methane concentration have been largely eliminated, the deviations in the model simulations are mainly due to errors in the surface methane fluxes. After optimization of both channels, the atmospheric CTM is run again from the unoptimized initial methane concentration of the current cycle, ensuring mass conservation of methane during the inversion process, to the start of the next cycle. The dual-pass procedure is then repeated until the entire inversion is completed.

## Supplementary Material

nwae223_Supplemental_File
