# Supplementary material for: Slowdown in China's methane emission growth
Source: Natl Sci Rev. 2024 Jun 26;11(8):nwae223. doi: 10.1093/nsr/nwae223 (PMC11389614; doi:10.1093/nsr/nwae223)
Supplement: nwae223_Supplemental_File [file nwae223_supplemental_file.docx]

Supplemental Information for

**Slow Down in China’s Methane Emission Growth**

Min Zhao^1^, Xiangjun Tian^1,2*^, Yilong Wang^1*^, Xuhui Wang^3^, Philippe Ciais^4^,

Zhe Jin^1,5^, Hongqin Zhang^6^, Tao Wang^1^, Jinzhi Ding^1^, Shilong Piao^1,5^

1 State Key Laboratory of Tibetan Plateau Earth System, Resources and Environment (TPESRE), Institute of Tibetan Plateau Research, Chinese Academy of Sciences, Beijing, 100101, China

2 University of Chinese Academy of Sciences, Beijing, 101408, China

3 Sino-French Institute for Earth System Science, College of Urban and Environmental Sciences, Peking University, Beijing, 100871, China

4 Laboratoire des Sciences du Climat et de l’Environnement, LSCE/IPSL, CEA-CNRS-UVSQ, Université Paris-Saclay, Gif-sur-Yvette, 91191, France.

5 Institute of Carbon Neutrality, College of Urban and Environmental Sciences, Peking University, Beijing, 100871, China

6 Institute of Atmospheric Physics, Chinese Academy of Sciences, Beijing, 100029, China

Correspondence to: Xiangjun Tian (tianxj@itpcas.ac.cn) and Yilong Wang [(wangyilong@itpcas.ac.cn)](mailto:(wangyilong@itpcas.ac.cn))

**This PDF file includes:**

Supplementary Texts

Supplementary Figures S1 to S24

Supplementary Tables

**Supplementary Text**

**TCCON data**

TCCON (Total Carbon Column Observing Network) measurements are an important source of validation for satellite observations and model simulations, including methane concentrations from the GOSAT satellites and GEOS-Chem simulations [1-3]. Unlike space-based instruments that measure reflected near-infrared sunlight by looking down from space, retrievals using TCCON spectra are least affected by aerosols, air-mass uncertainties, or changes in land-surface properties, because TCCON instruments measure direct incident sunlight from the ground. TCCON data can therefore be used as a standard for transfer between satellite observations and in situ networks [4]. Due to their high quality, TCCON data are one of the best real-world data sources for validating model simulations of methane concentrations. In this study, we used the data from 26 TCCON stations (Fig. S19) from 1 January 2011 to 31 December 2021 to evaluate modelled atmospheric CH_4_ mole fractions driven by prior and posterior flux (Fig. S20).

**ObsPack data**

ObsPack (Observation Package) is a data product that gathers greenhouse gas observations from various platforms like surface stations, aircraft campaigns, tall towers, or ships. It compiles and distributes these observations in a consistent and well-documented format, aiming to support carbon cycle modeling studies [5]. For our study, we utilized surface flask observations from obspack_ch4_1_GLOBALVIEWplus_v5.1_2023-03-08. These flask observations are fully independent from the TCCON data satellite observations. Surface flask observations are collected about once a week at most stations. We divided the globe into seven regions: North America, South America, Europe, Africa, East Asia, Southeast Asia, and Australia (Fig. S21). Within each region, three stations whose time series are relatively completed were selected as representatives. We used the surface flask observations from these 21 sites (Fig. S21) to evaluate modelled atmospheric CH_4_ mole fractions driven by prior and posterior flux (Fig. S22).

**Evaluation of methane fluxes using independent data**

We perform posterior simulations using the prior (E2) and posterior methane emissions from GONGGA-CH_4_ and evaluated the modelled XCH_4_ with independent data from the TCCON and ObsPack observations. These data were not assimilated into the inversion system. The modelled CH_4_ mole fractions were sampled at the locations and times corresponding to the observations, and we calculated the mean bias and root mean square error (RMSE) values during the whole period from 2011 to 2021.

Figure S20 shows the evaluation against TCCON observations. The modelled XCH_4_ driven by prior fluxes have a global mean bias and RMSE of -61.4 ppb and 64.9 ppb, and the biases are exacerbating with time. After inversion, the modelled XCH_4_ driven by posterior fluxes have much smaller biases and RMSE, with a global mean of 9.7 ppb and 13.2 ppb. Furthermore, the biases across all sites are near zero during the whole period without significant trends.

Figure S22 shows the evaluation against ObsPack surface observations. The modelled CH_4_ mole fractions driven by prior fluxes have a global mean bias and RMSE of -53.0 ppb and 59.8 ppb, and the biases are increasing with time. After inversion, the modelled XCH_4_ driven by posterior fluxes have much smaller biases and RMSE, with a global mean of 14.9 ppb and 21.9 ppb. Furthermore, the biases across all sites are near zero during the whole period without significant trends.

In conclusion, the prior fluxes were underestimated with negative biases, and the posterior fluxes corrected them by assimilating GOSAT XCH_4_ data. These two evaluations against TCCON and surface observations confirm that our posterior fluxes are well optimized without significant biases during the whole period at both global and regional scales.

**Comparisons of methane emissions in GONGGA-CH_4_ with GCP**

Methane in the Chinese region is mainly distributed in Northeast, North, East, South and some Southwest China. The range of wetlands in the Chinese region is small, and the methane emissions are mainly caused by anthropogenic sources. Comparing with the spatial distribution of other inversion systems (partly systems), the methane fluxes simulated by GONGGA-CH_4_ are reasonably distributed (Fig. S24). The differences in the different inversion inventories are due to the differences in the priori fluxes, the assimilated observations and the atmospheric transport models used in each inversion system. We employed three distinct experimental setups with GONGGA-CH_4_ framework, each incorporating different prior fluxes, resulting in substantial discrepancies among the three experiments. Specifically, The prior in E1 from EDGARv6, E2 from EDGARv7, and E3 from the posterior results of the CarbonTracker Europe (CTE) system. Examination of the outcomes reveals that the 'burning' component in E1 and E2 exclusively encompasses biomass burning, with the prior information sourced from Global Fire Emissions Database v4 (GFED4) fire data, consequently yielding lower values in the 'biomass and biofuel burning' category. This result underscores the pivotal role of the discrepancies in ratios within the prior information in influencing the corresponding ratios in the posterior outcomes. From the perspective of posterior results, the E3 experiment utilizing the CTE posterior exhibits closer alignment with the results from the Global Carbon Project (GCP), both in terms of total quantities and the proportions of distinct components. The utilization of the CTE posterior is noteworthy, as it is a reanalysis outcome already assimilating station data. This observation signifies that, from an inversion standpoint, superior prior information can better encapsulate the accuracy of posterior distributions. Overall, our ensemble results are very close to the GCP averages results (Fig. S2).

**Coal mine rectification policy**

In 2009, Chinese issued the "Notice on Organizing Special Rectification of Gas in Small Coal Mines" (Development and Reform Energy Energy [2009] No. 889). The special rectification of gas in small coal mines targets coal mines with an annual production capacity of 300,000 tons yr^-1^ or less, including various types of coal mines involved in infrastructure construction, technological upgrading, resource integration, and normal production. Different provinces have different timelines for implementation (i.e. Hunan Province within one and a half years, and Lanzhou City within three years). The rectification process is divided into three stages: self-inspection and self-correction by enterprises, centralized consultation and rectification, and inspection, acceptance, and consolidation of achievements. This is mainly reflected in the consolidation of different coal mines, the closure of small mines and the elimination of inefficient coal production, and the consolidation of large coal mines to better regulate their operations. As the structural reform of the coal supply advances, a large number of coal mines with severe disasters and depleted resources have been gradually closed and phased out. The total number of coal mines in the country has decreased from 37,000 at the beginning of this century to 5,268 in 2019. After the closure of coal mines, goafs, mining disturbance unloading zones, and original coal seams often contain a significant accumulation of coal mine gas, posing significant risks of accidents.

**Bottom-Up inversions of Rice emissions**

A bottom-up approach to inverse methane emissions from rice was used to conduct a sensitivity test to verify the effect of straw return policy on methane emissions. This method is from Yan et al. [6]. The following equation was applied to each type rice to calculate methane emission from rices.

$E_{\text{rice}}=\sum_{i} \sum_{j} \sum_{k} (EF_{ijk}A_{ijk}L_{ijk})$,

where *i* is different types of ice, including early rice, middle rice, and late rice. According to the data in China National Bureau of Statistics (Fig. S10), the classification of rice types obtained here is early rice, middle and one-season late rice, and two-season late rice. The *j* is water regime (intermittent irrigation and continuous flooding), here we assumed 66.7% and 33.3% for intermittent irrigation and continuous flooding, respectively, following [7]. The parameter *k* is with or without organic input, that is refer to whether to implement the policy of returning straw to the field. *EF_ijk_* is the rice emission factor, whose values were taken from Yan et al. [6]. *A_ijk_* is the rice cultivation area, which are taken from the China National Bureau of Statistics for different types of rice. *L_ijk_* is the length of rice-growing period.

**Sensitivity tests of hyperparameters of the GONGGA-CH_4_**

We performed a series of sensitivity tests to assess the uncertainty of inversions associated with the inversion configurations including the length of assimilation window and localization radius. In these sensitivity tests, the prior fluxes and other configuration are kept the same as E2 experiment, but only using varying lengths of assimilation window and localization radius. For the length of assimilation window, 14 days and 28 days are tested, and 1000 km and 2000 km are tested for the localization radius. Due to the significant time and computational demands of a full inversion, we test the impacts of these parameters for the year 2016. The national total emissions of the posterior methane fluxes from the sensitivity tests and the prior fluxes are shown in Fig. S18. The length of assimilation window and localization radius have marginal impacts on the national budget of China’s CH_4_ emissions. At the same time, the values of posterior fluxes associated with different choices of assimilation window and localization radius are much smaller than the correction of posterior fluxes, confirming that these hyperparameters have small impacts on the inverted fluxes.

**Impacts of atmospheric oxidization and dual-pass inversion strategy**

Some studies suggested that global OH can be buffered against short-term anthropogenic and natural perturbations, and thus has a small interannual variability [8]. However, recent top-down estimates based on observations of 1,1,1-trichloroethane (CH_3_CCl_3_) and the C^13^/C^12^ ratio in atmospheric methane suggested that global OH decreased by 0.5%-1% per year since 2003 [9, 10]. In addition, the global OH was found to be 1.6%-1.8% below the 2019 level in 2020 [11] and 3% below the 2019 level in 2021 [12]. In GONGGA-CH_4_ system, we used a climatology OH field to simulate the atmospheric oxidization with no trend and rely on the concentration channel in the dual-pass framework to correct for the impacts of ignorance of interannual variability of OH fields. To verify its validity, we further conduct a sensitivity test with varying OH fields and compare the results with the inversions that use constant OH fields.

We choose the E2 experiment as the reference inversion. Then we configure a new inversion (E2_OHchanged) by scaling the OH fields to achieve a decreasing trend of 1% per year from 2015 to 2019, a reduction of 1.6% in 2020 compared to 2019, and a reduction of 3% in 2021 compared to 2019, and keeping the other configurations the same as E2. The E2_OHchanged inversion was run since 2015 using the same initial condition on Jan 1^st^, 2015 as E2 and with the first year as a spin-up time.

Figure S23 shows the comparison between the posterior estimates of China’s CH_4_ emissions from E2 and E2_OHchanged. The interannual variability were quite similar between the two inversions. As a result, China’s CH_4_ emissions from E2_OHchanged experiment also shows a slowdown of increase, corroborating that our results were not biased and the dual-pass strategy is effective in alleviating potential bias from the use of constant OH.

**Supplementary Figures**


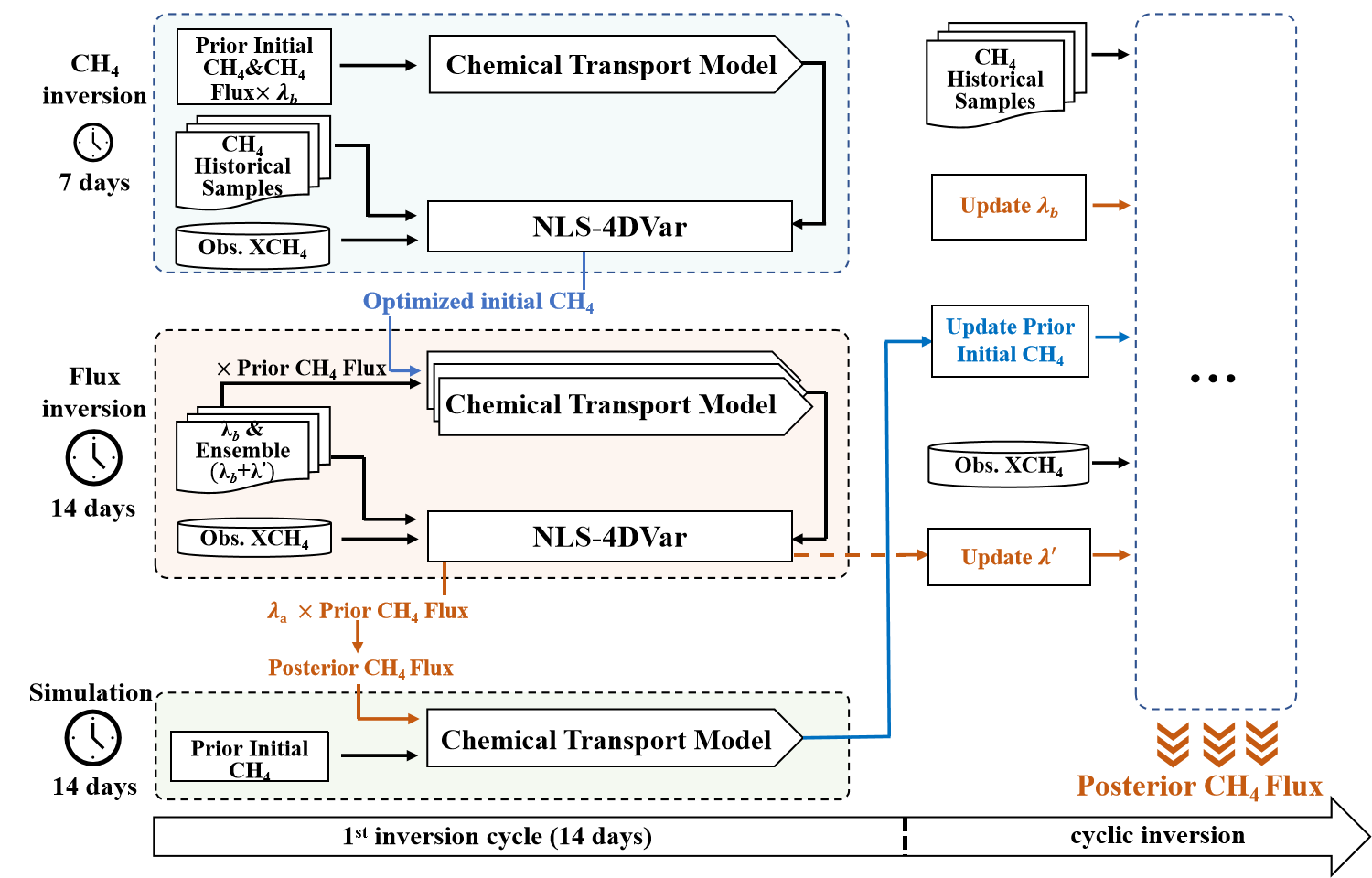


**Fig. S1**. The flowchart of the GONGGA-CH_4_ system.

**
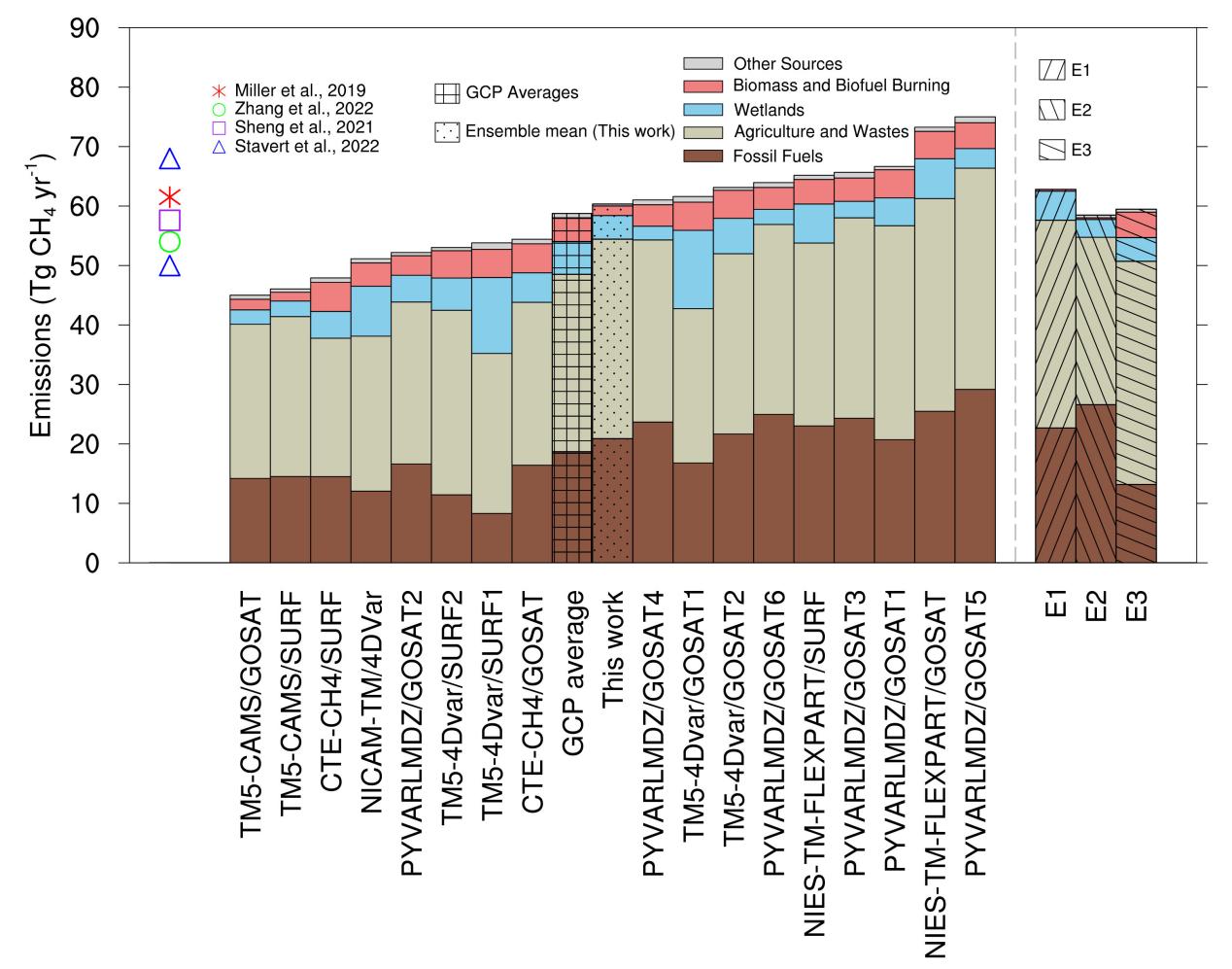
Fig. S2**. Annual mean methane emissions over China from GONGGA-CH_4_ in 2017 and Global Carbon Project (GCP) in 2017, and previous estimates from [13-16] are provided. Boxes represent GCP averages and dots represent GONGGA-CH_4_ results. E1, E2, and E3 represent three experiments with different priors.


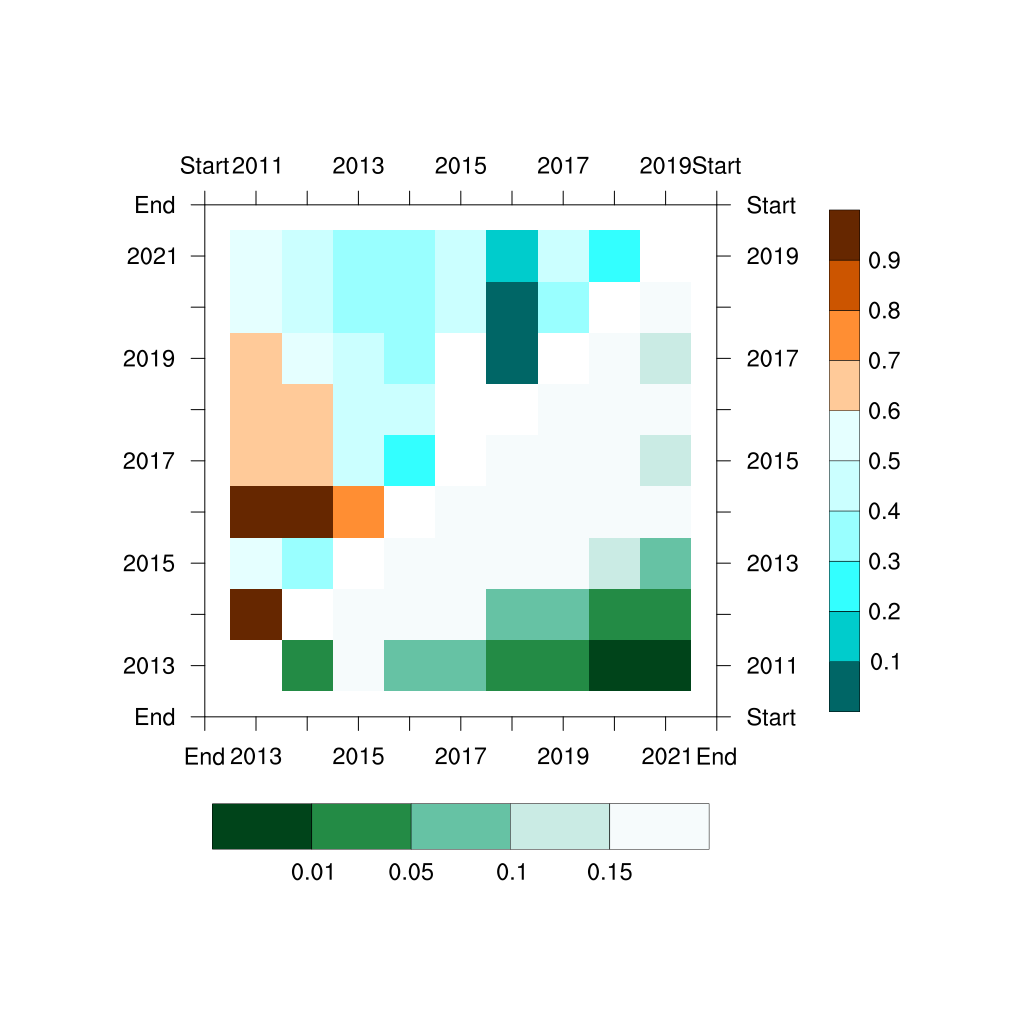


**Fig. S3**. Matrices of China’s annual methane emissions trend. The matrices of the methane emissions trend during periods with different combinations of start and end year are shown in the upper left part and the corresponding p-values are shown in the lower right part.


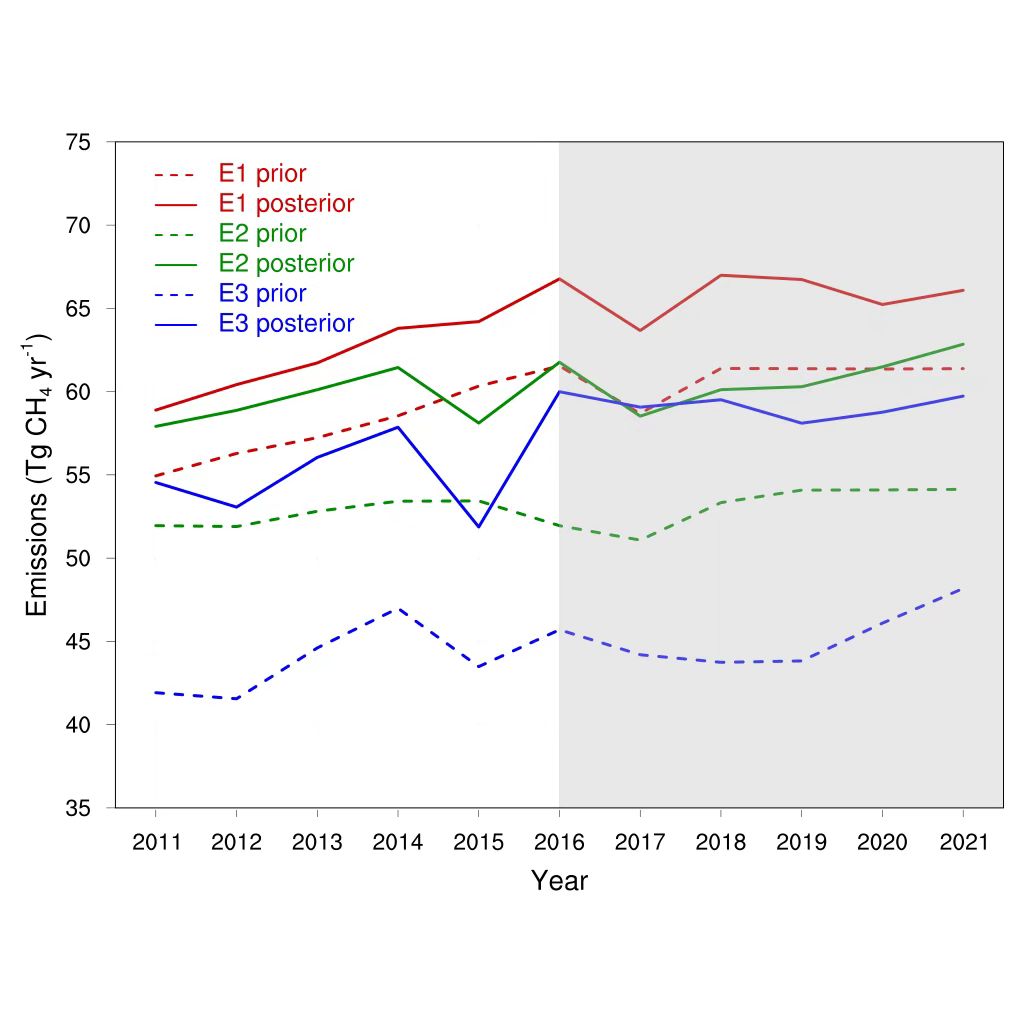


**Fig. S4.** Prior (dashed lines) and posterior (solid lines) methane emissions in China from three inversions using different configurations of prior information.


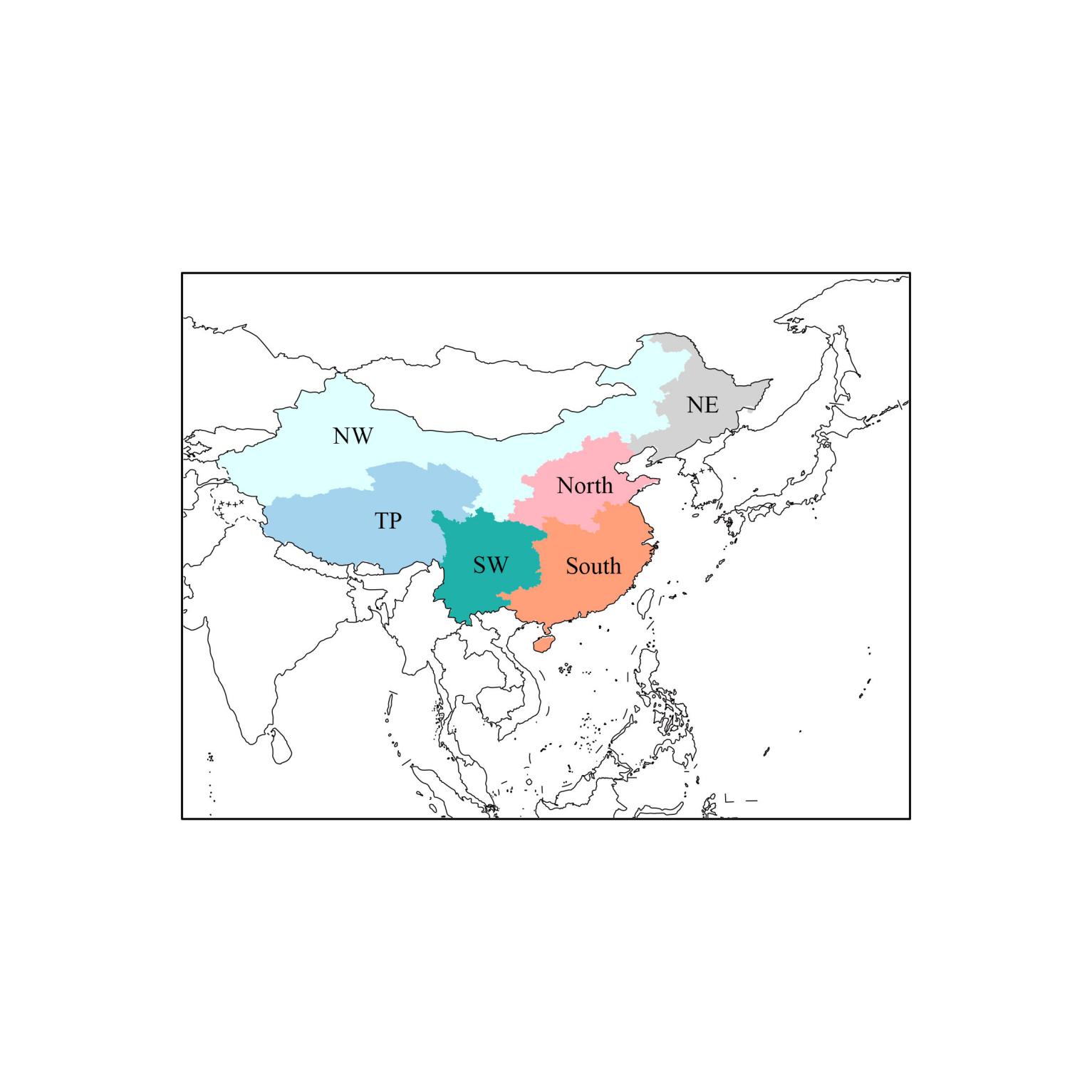


**Fig. S5.** The six regions used in this study. The NW represents Northwest, the NE represents Northeast, the SW represents Southwest, and the TP represents Tibetan Plateau.


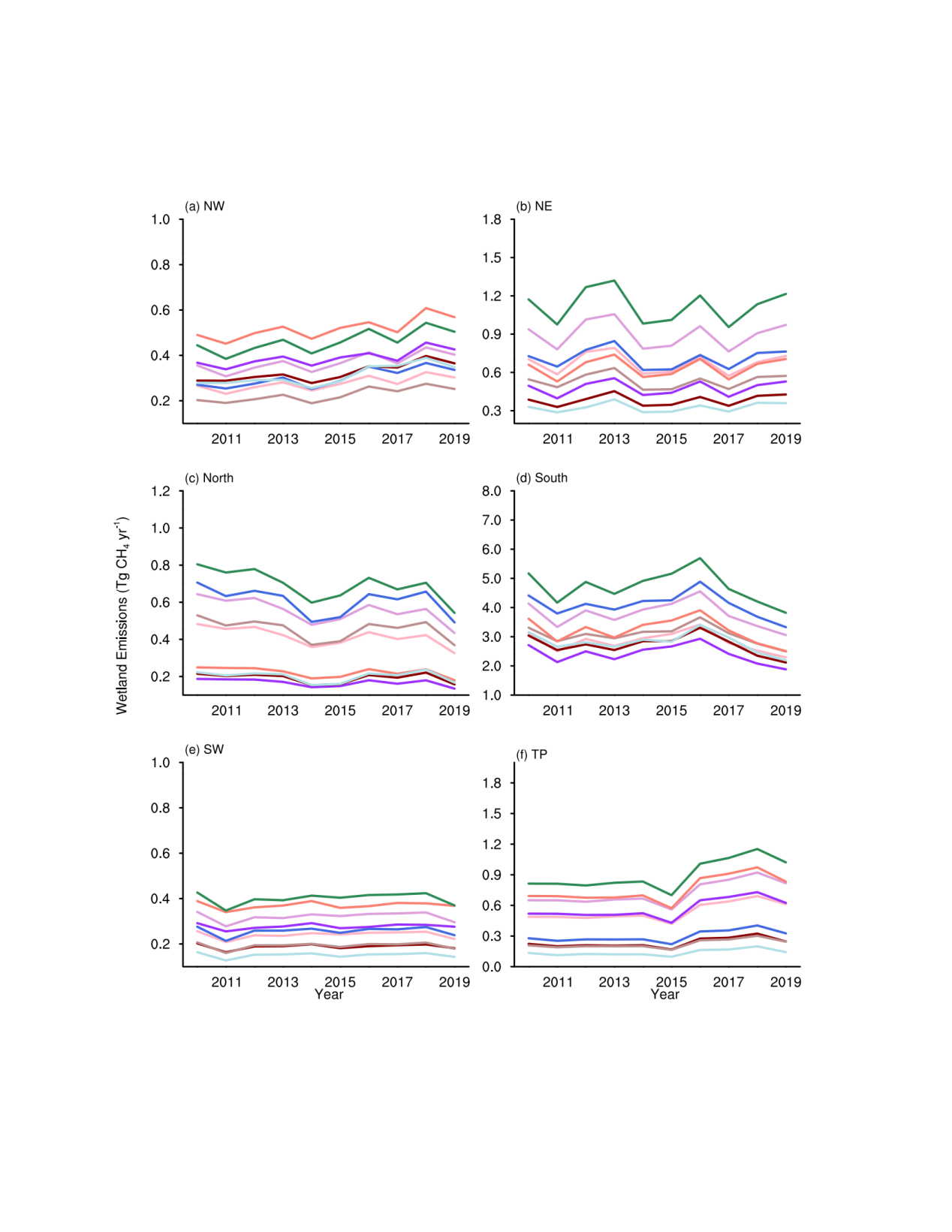


**Fig. S6.** Wetland methane emissions (Tg CH_4_ yr^-1^) from different regions over China in WetCHARTs dataset (2011-2019). The different lines in plots represent the results of different models.


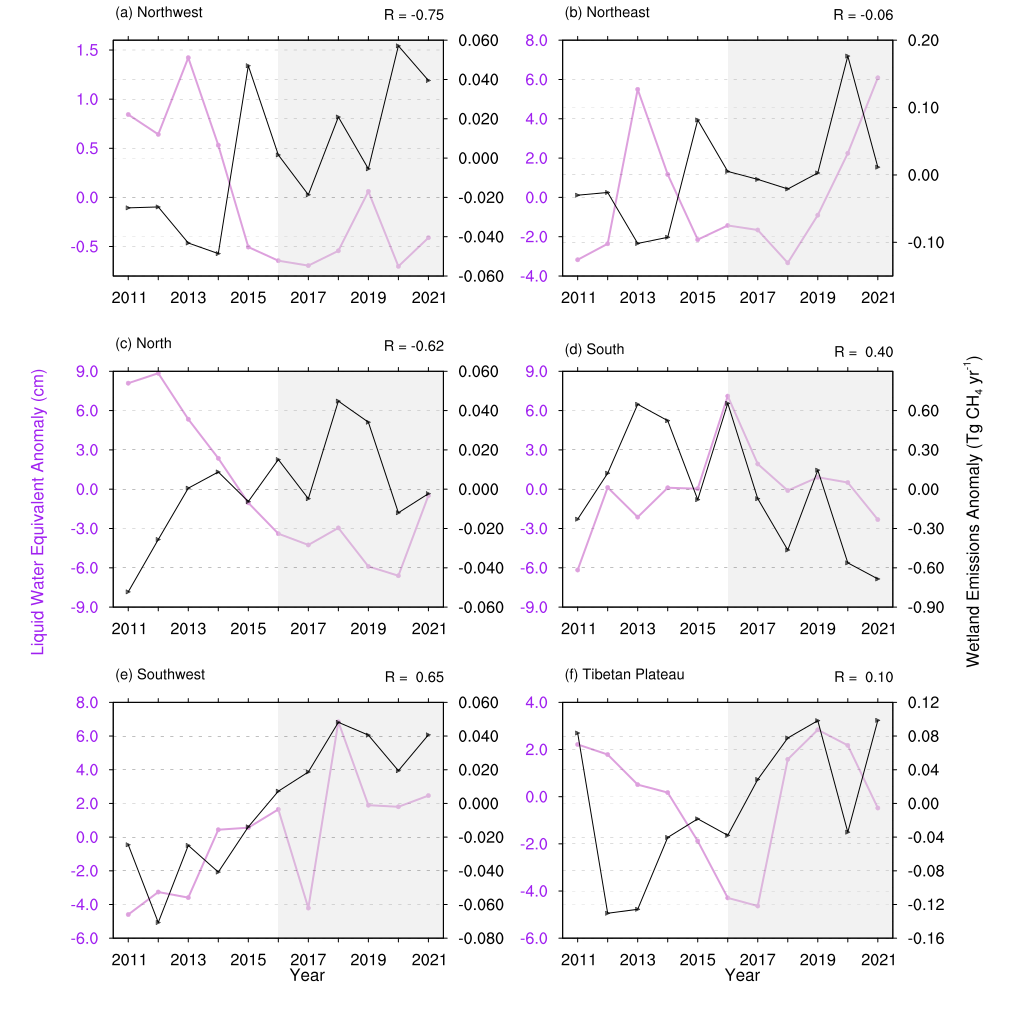


**Fig. S7**. The relationship between anomalies in liquid water equivalent height observed by GRACE-FO satellite and wetland emissions for different regions in China.


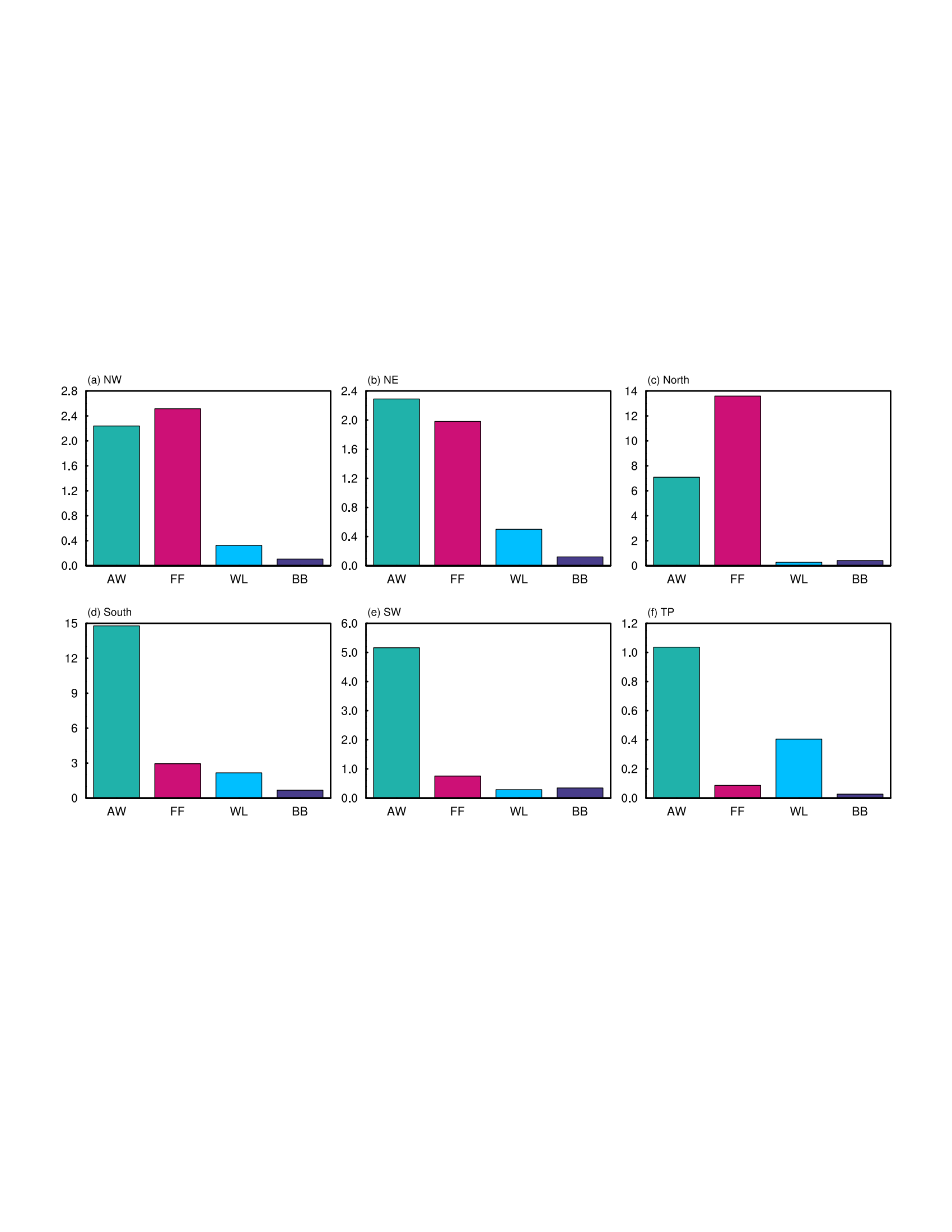


**Fig. S8**. Bar charts for 4 main methane emissions in different regions of China. Detailed data and standard deviations for the three experiments are shown in Table S2. AW: Agriculture and Wastes; FF: Fossil Fuels; WL: Wetlands; BB: Biomass and Biofuel Burning.


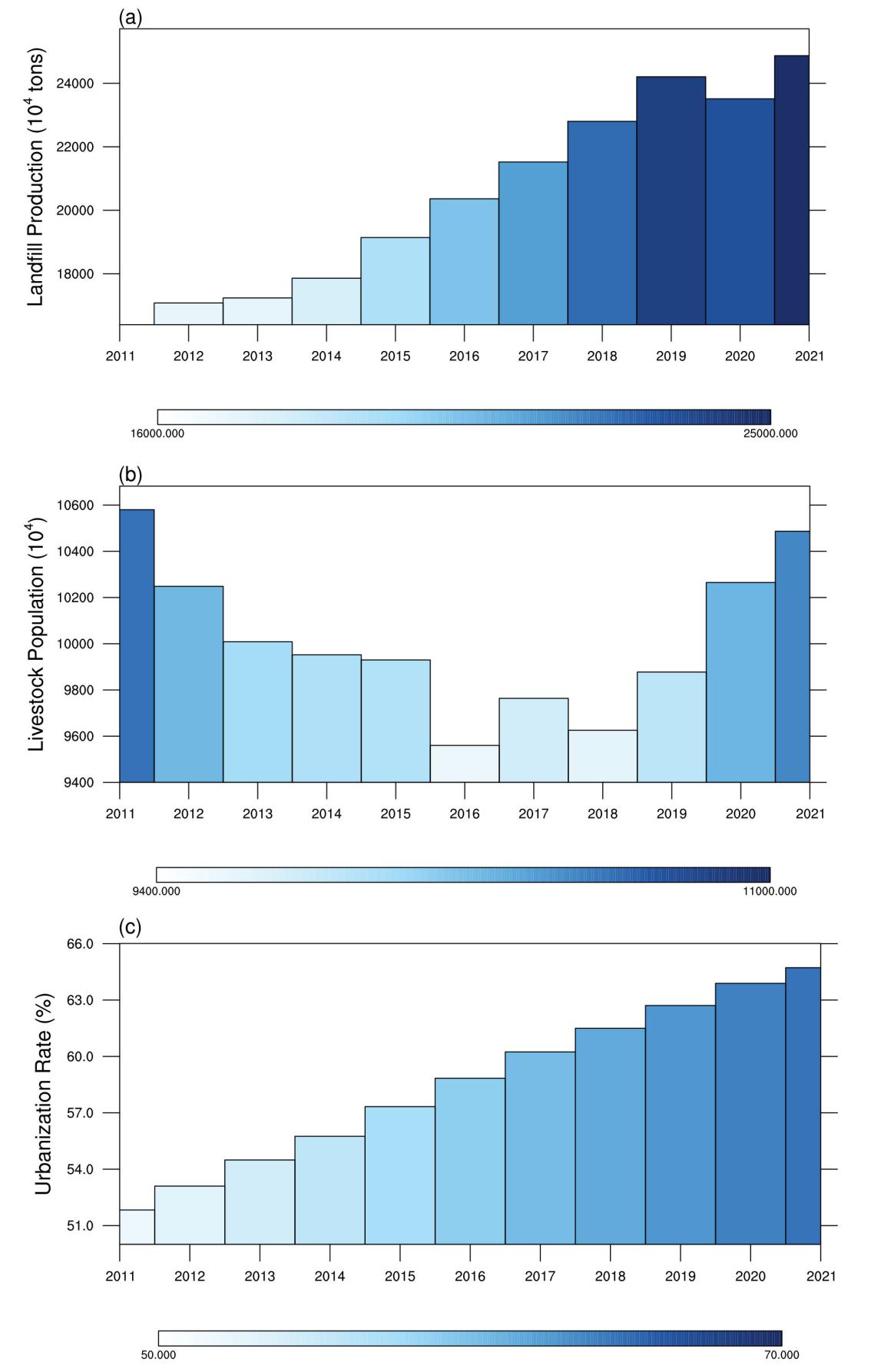


**Fig. S9**. The time series of annual mean of Landfill Production (10^4^ tons), Livestock Population (10^4^), and Urbanization Rate (%).


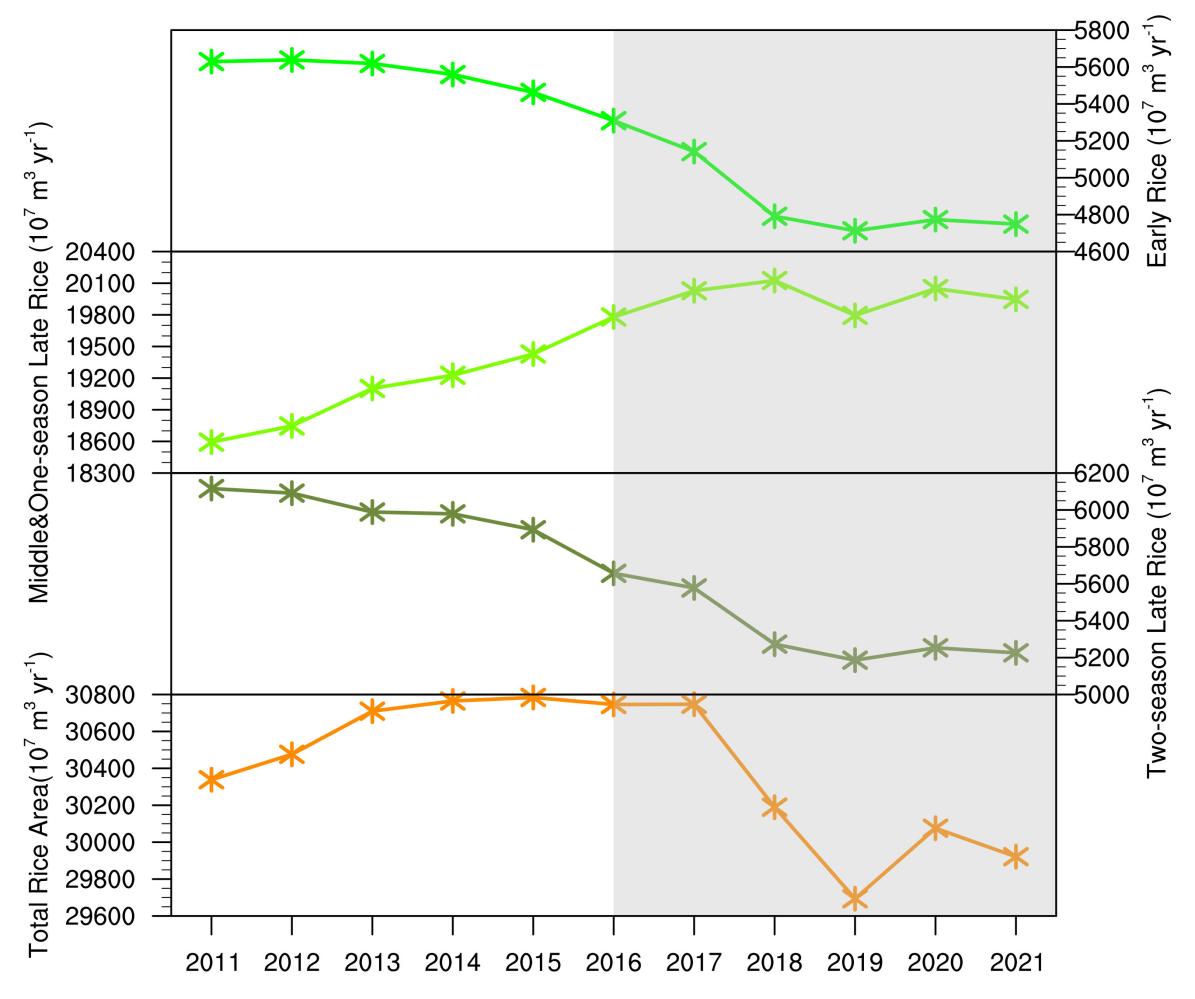


**Fig. S10**. The time series of annual mean of Early Rice, Middle and one-season Late Rice, two-season Late Rice and total rice Areas (unit: 10^7^ m^3^ yr^-1^) in 2011-2021.


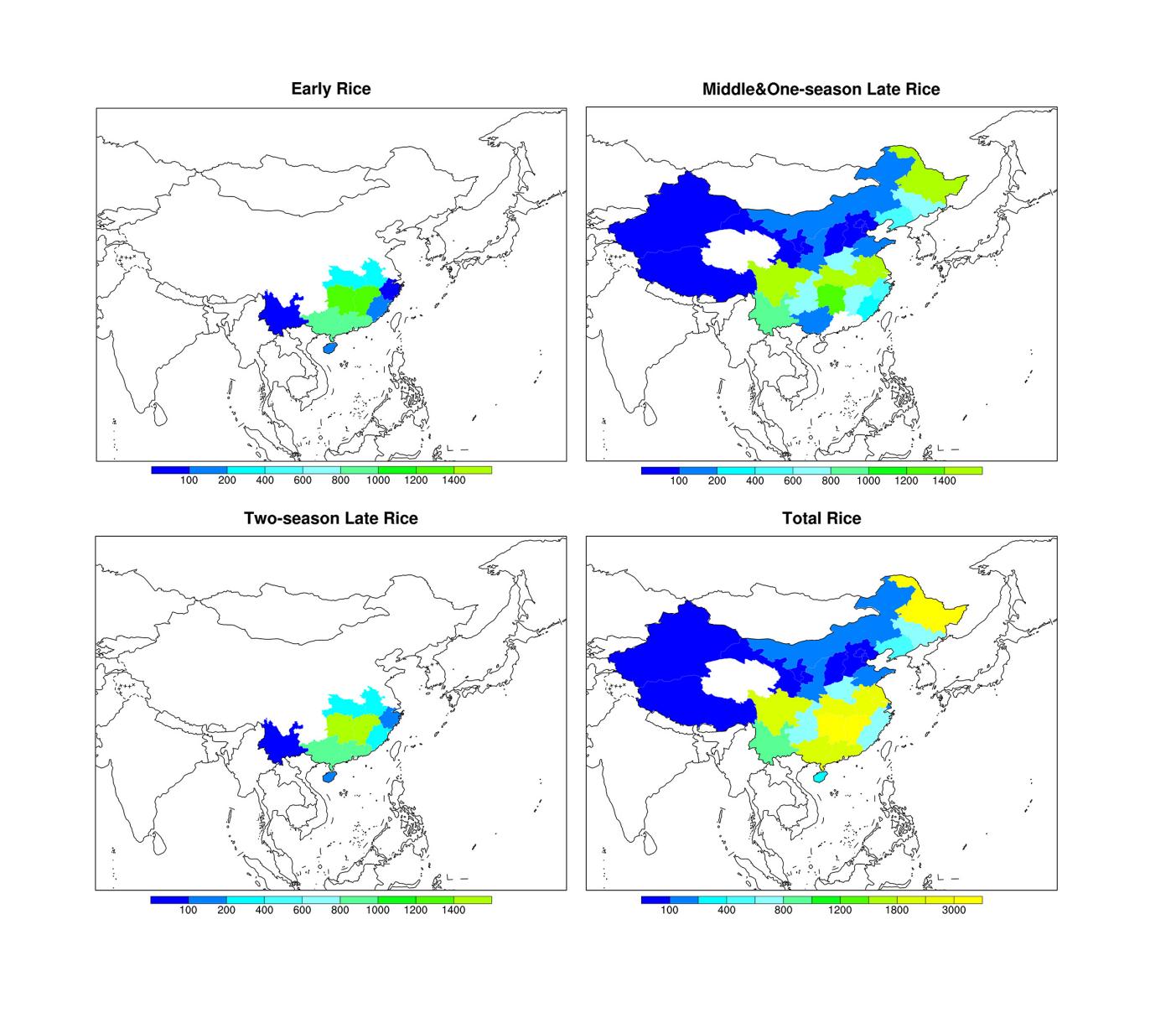


**Fig. S11**. The distribution of rice cultivation areas (unit: 10^7^ m^3^ yr^-1^) (average between the period 2011-2020) over China.


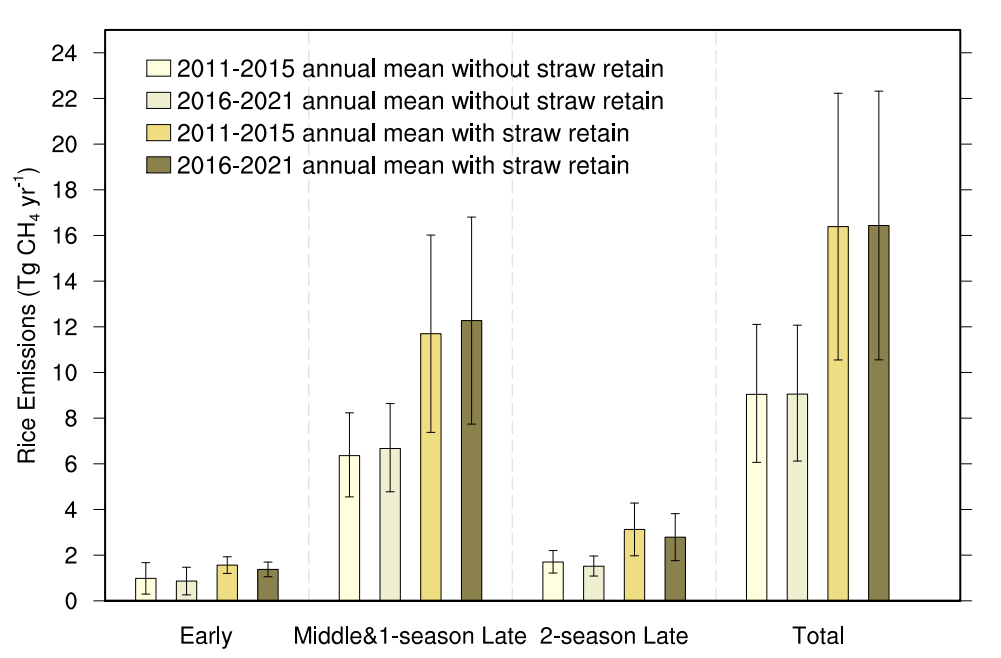


**Fig. S12**. The impact of straw return policy on methane emissions from different types of rice, including and excluding the straw return factor for two time periods. When determining whether or not to return straw to the field at both stages, we consider the ideal scenario in which the emission factor for straw return is either entirely disregarded or entirely taken into account.


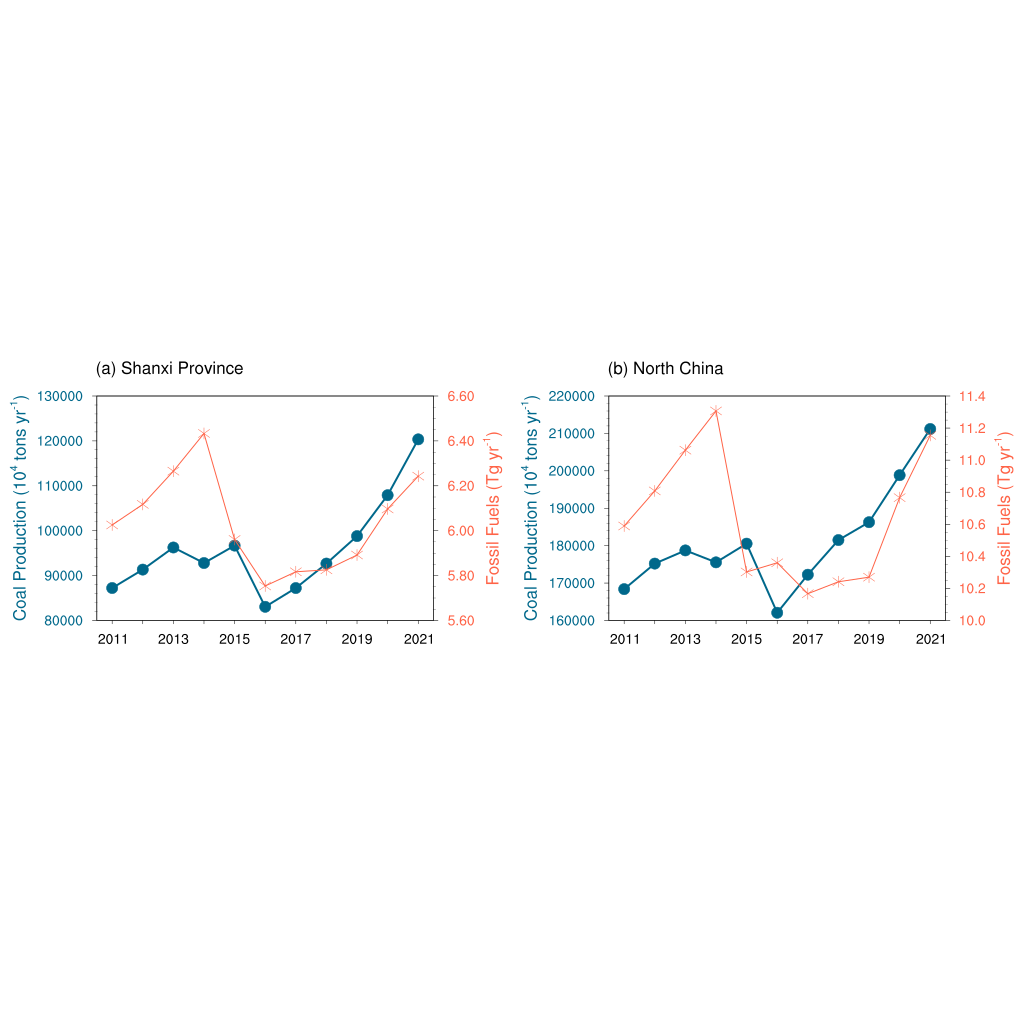


**Fig. S13**. Comparison of coal production and fossil fuel emissions in major coal-emitting Shanxi Province and North China. The North China includes Shanxi province, Hebei province, Shaanxi province, Shandong province, and Henan province.


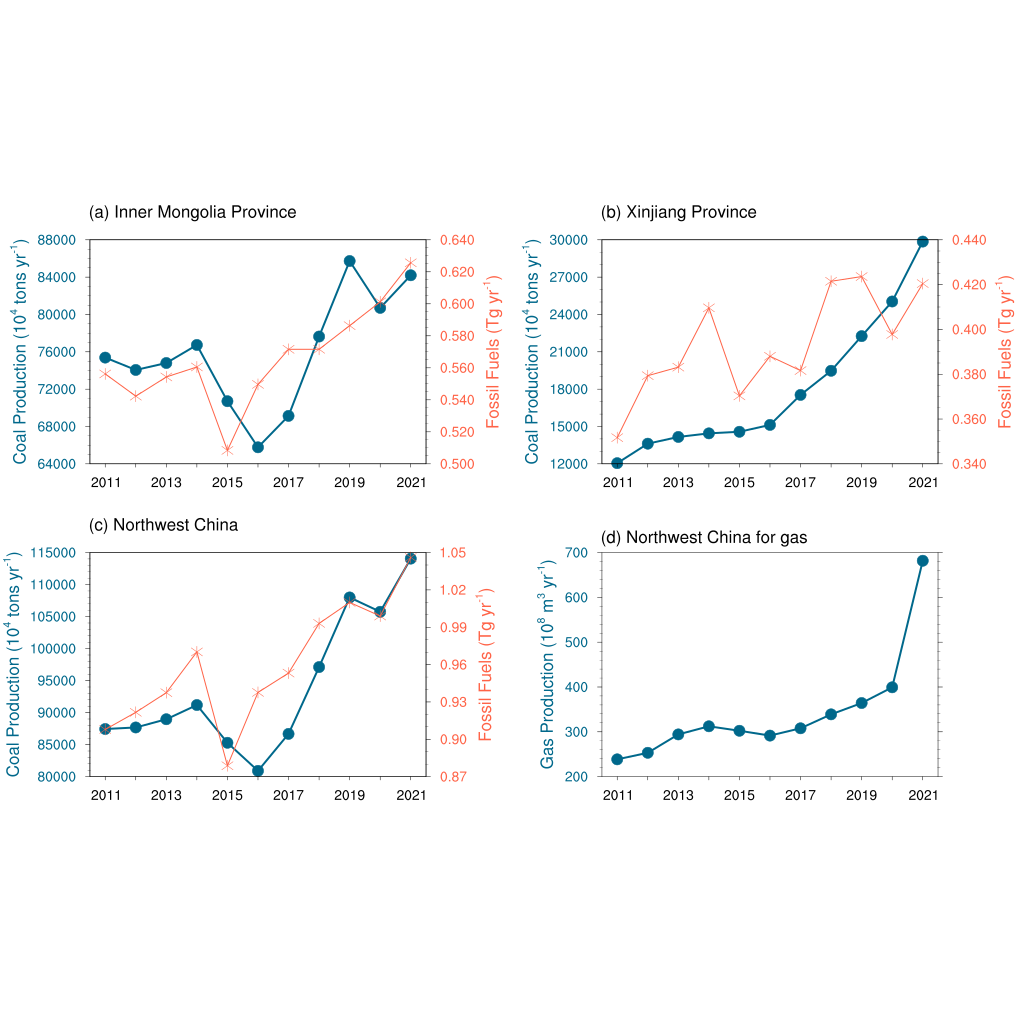


**Fig. S14**. (a-c) Comparisons of coal production and fossil fuel emissions in major coal-emitting Inner Mongolia Province, Xinjiang Province, and Northwest China. (d) The gas production in the Northwest China. The Northwest China includes Inner Mongolia Province, Xinjiang Province, Ningxia province, Gansu province.


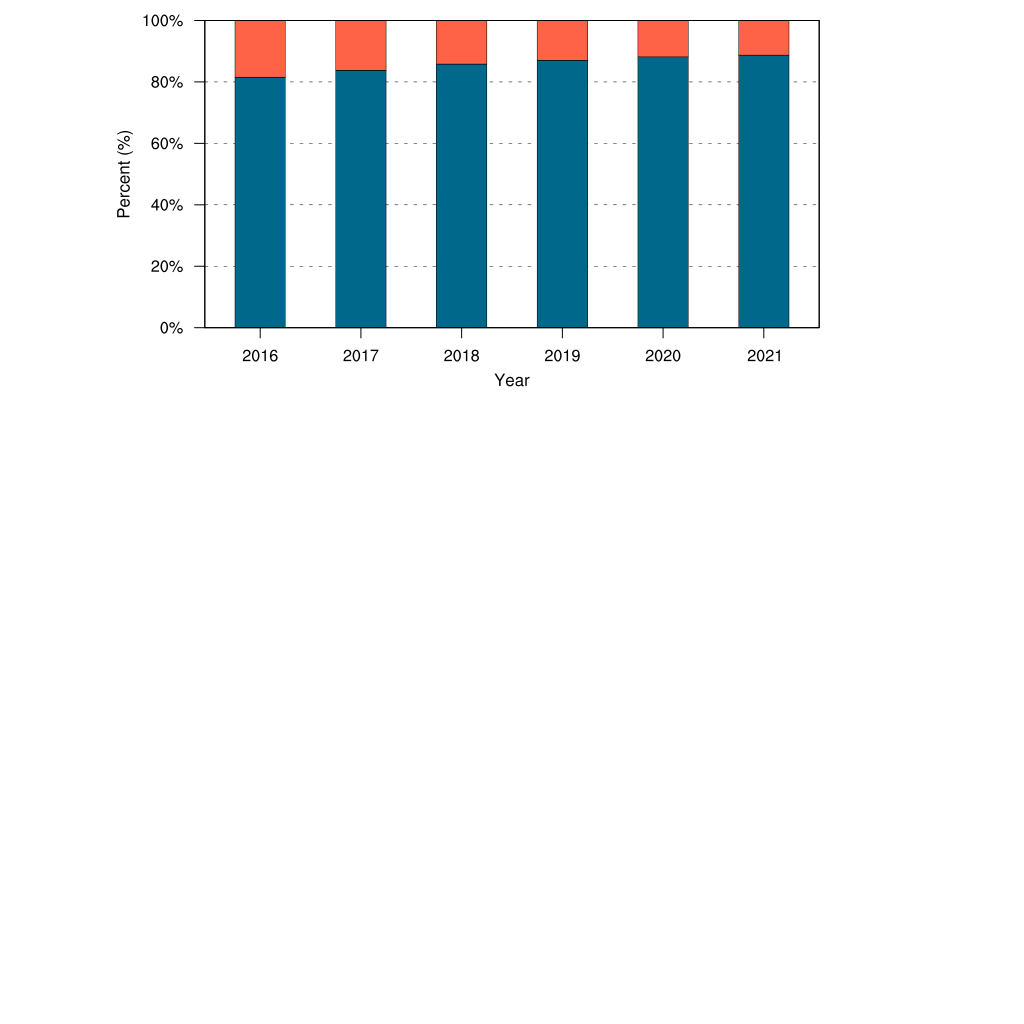


**Fig. S15.** Percent of coal production in the North and Northwest China (blue), and other regions (orange).


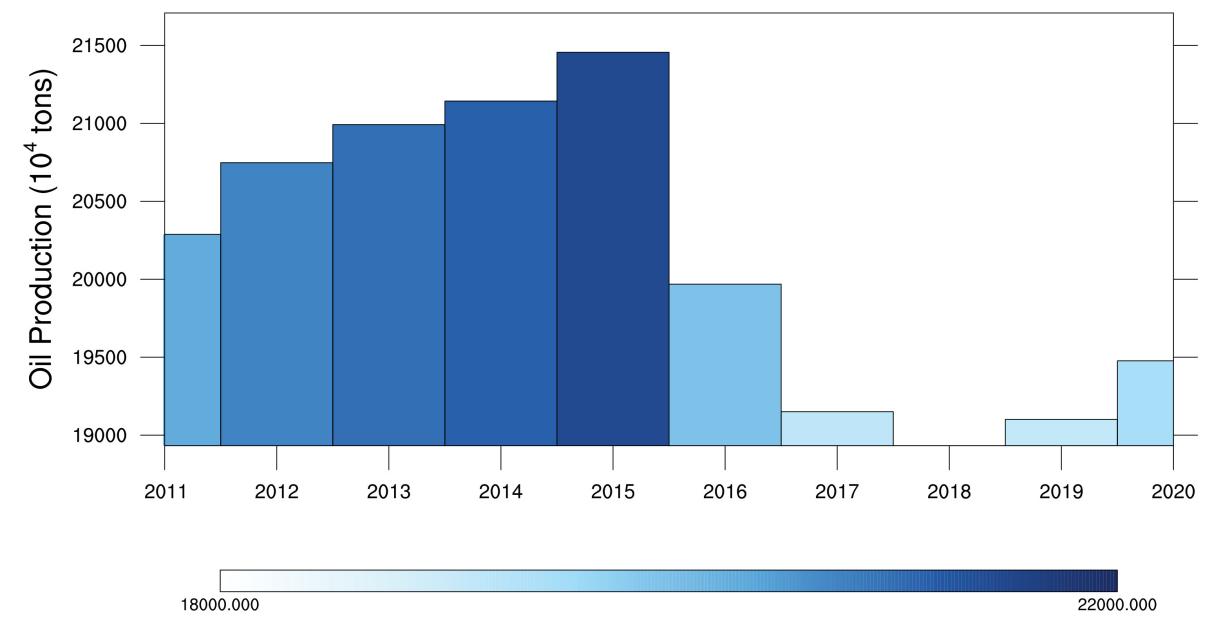


**Fig. S16.** Time series of annual mean of Oil Production (10^4^ tons yr^-1^) over China.


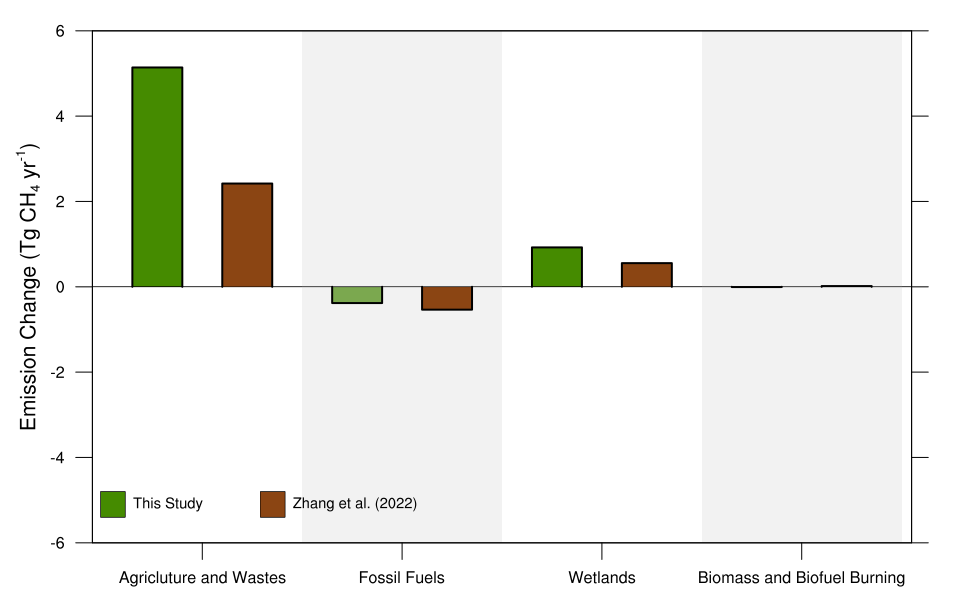


**Fig. S17**. Methane emission changes from 2011 to 2016 for different sectors. Green bars are results from our study, and brown ones are from Zhang et al. [15].


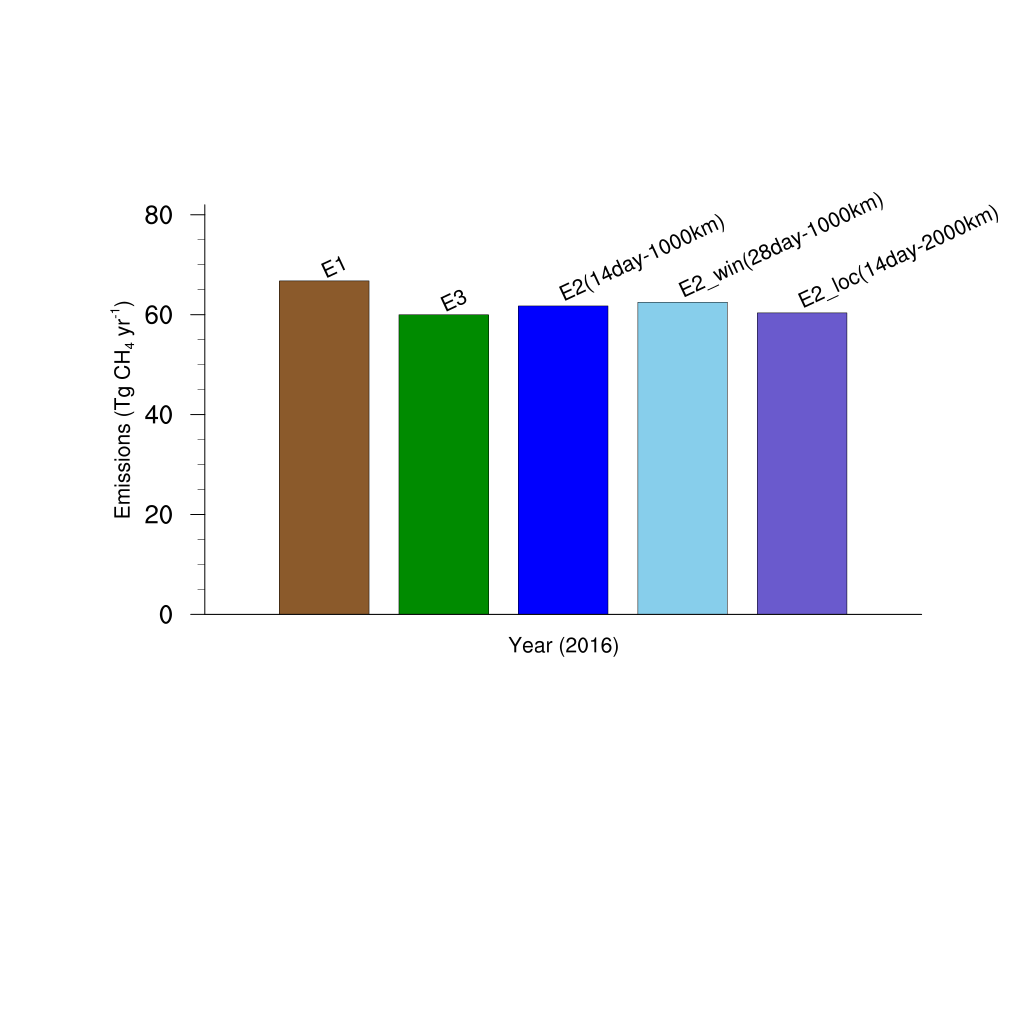


**Fig. S18**. Sensitivity test results from the year 2016. E1, E3, and E2 are the main experiments in our study; E2_win represents a sensitivity experiment altering the flux assimilation windows by 28 days compared to the E2 experiment by 14 days; E2_loc represents a sensitivity experiment for localization radius by 2000 km compared to E2 experiment by 1000 km.


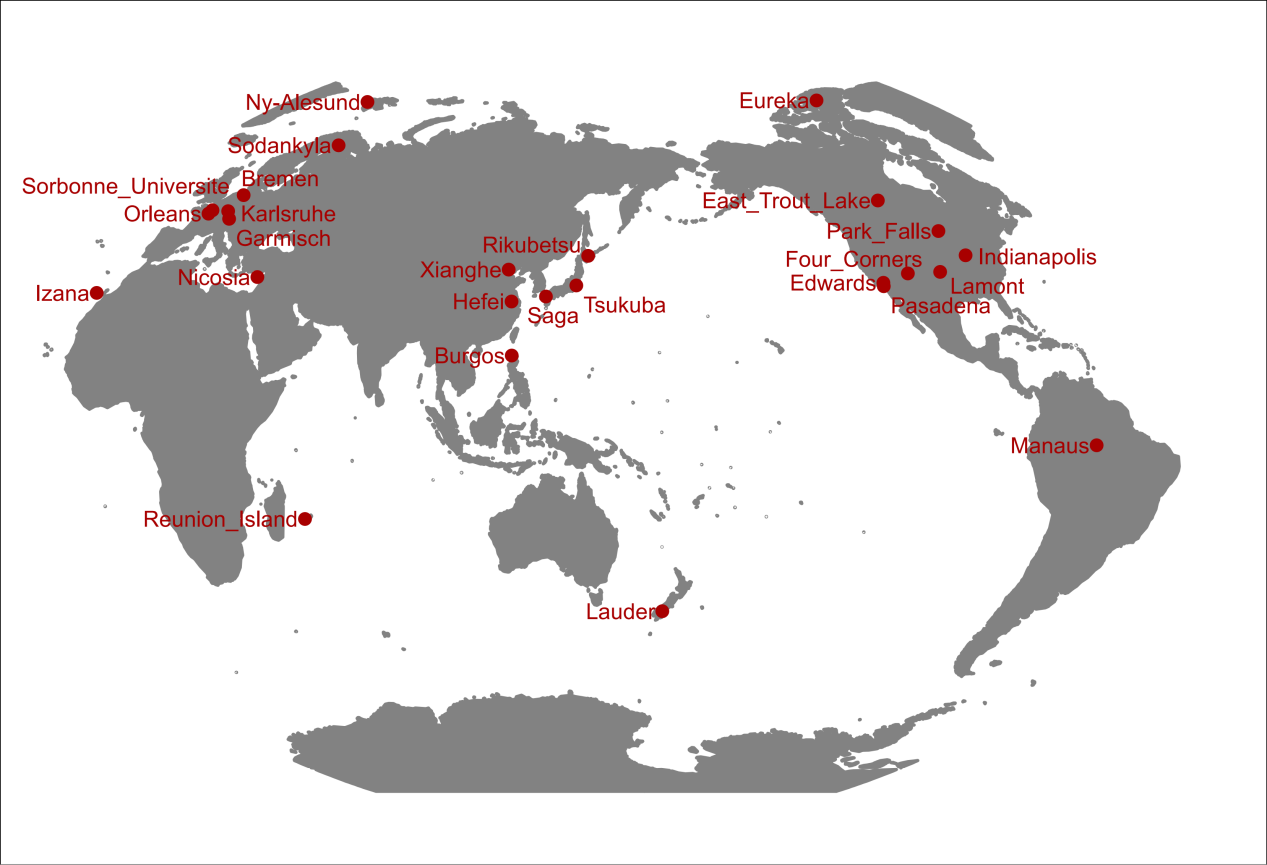


**Fig. S19**. The spatial distributions of TCCON sites used for evaluations.


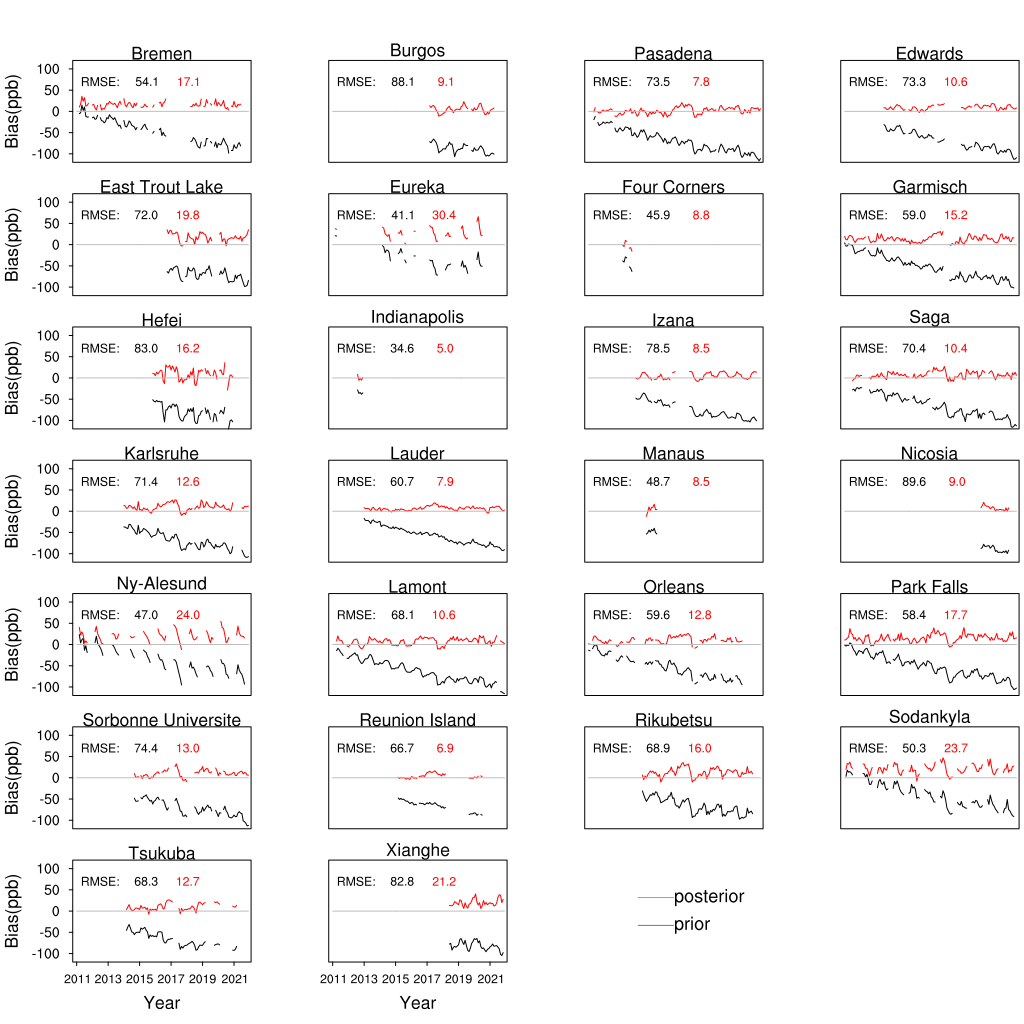


**Fig. S20**. Monthly biases and RMSE values of modelled XCH_4_ driven by prior and posterior fluxes at 26 TCCON sites.


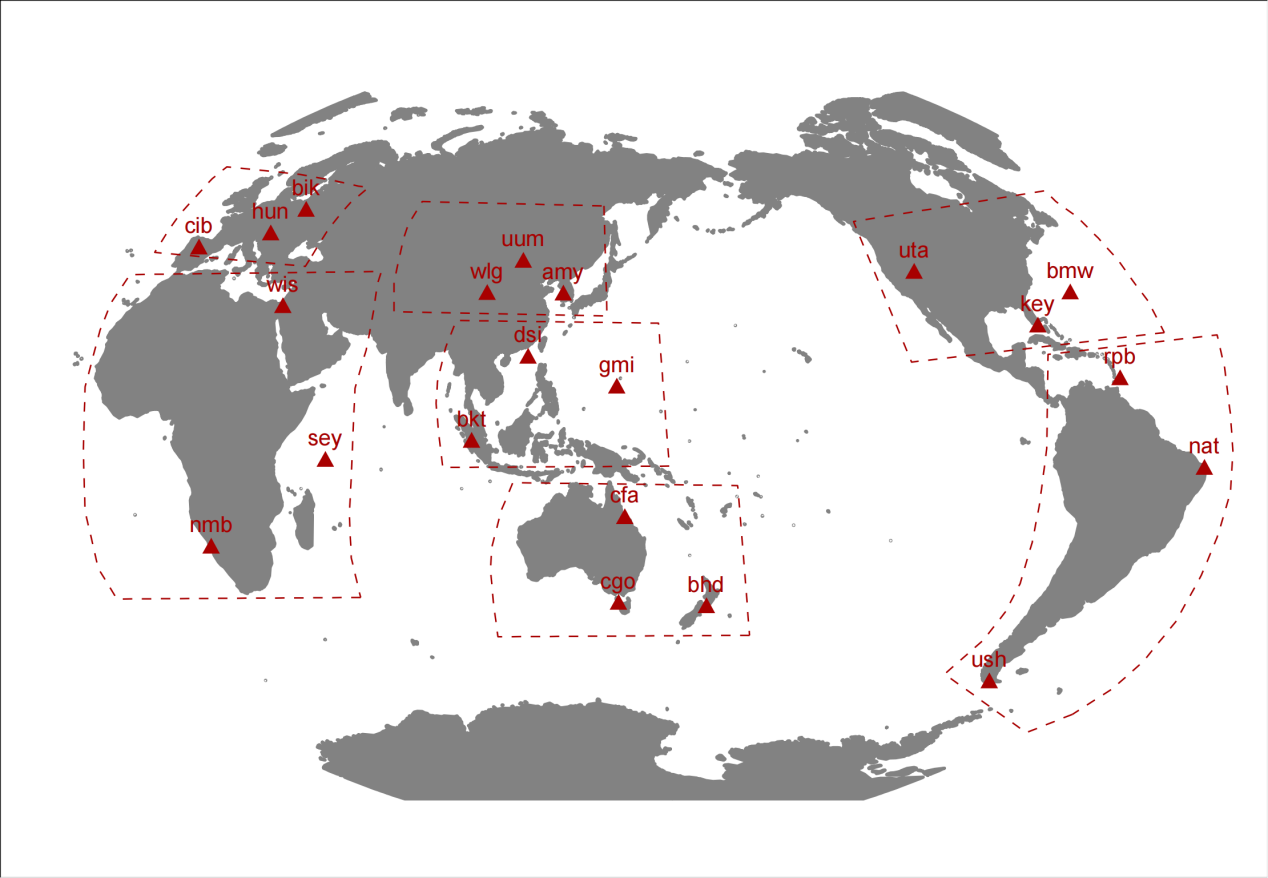


**Fig. S21**. The spatial distributions of ObsPack sites used for evaluations.


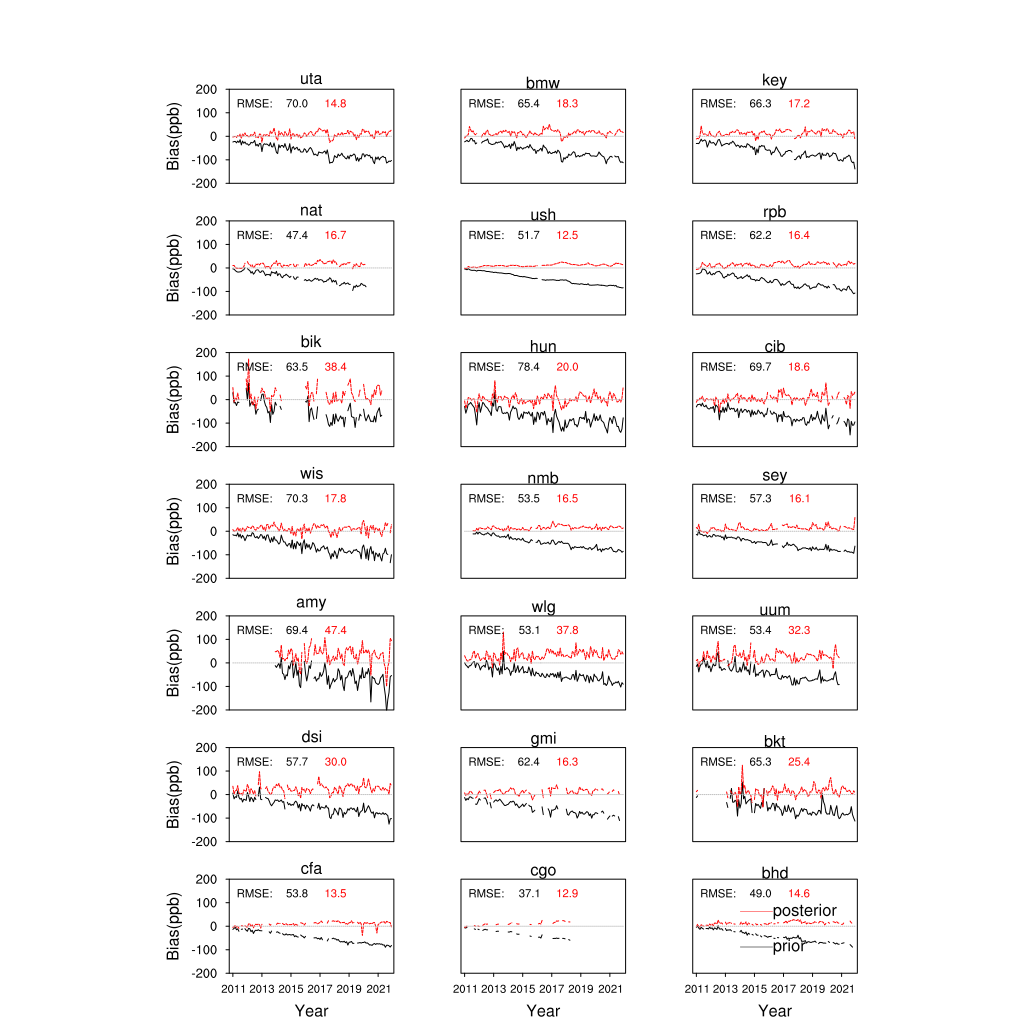


**Fig. S22**. Monthly biases and RMSE values of modelled CH_4_ driven by prior and posterior fluxes at 21 surface stations from the ObsPack dataset.


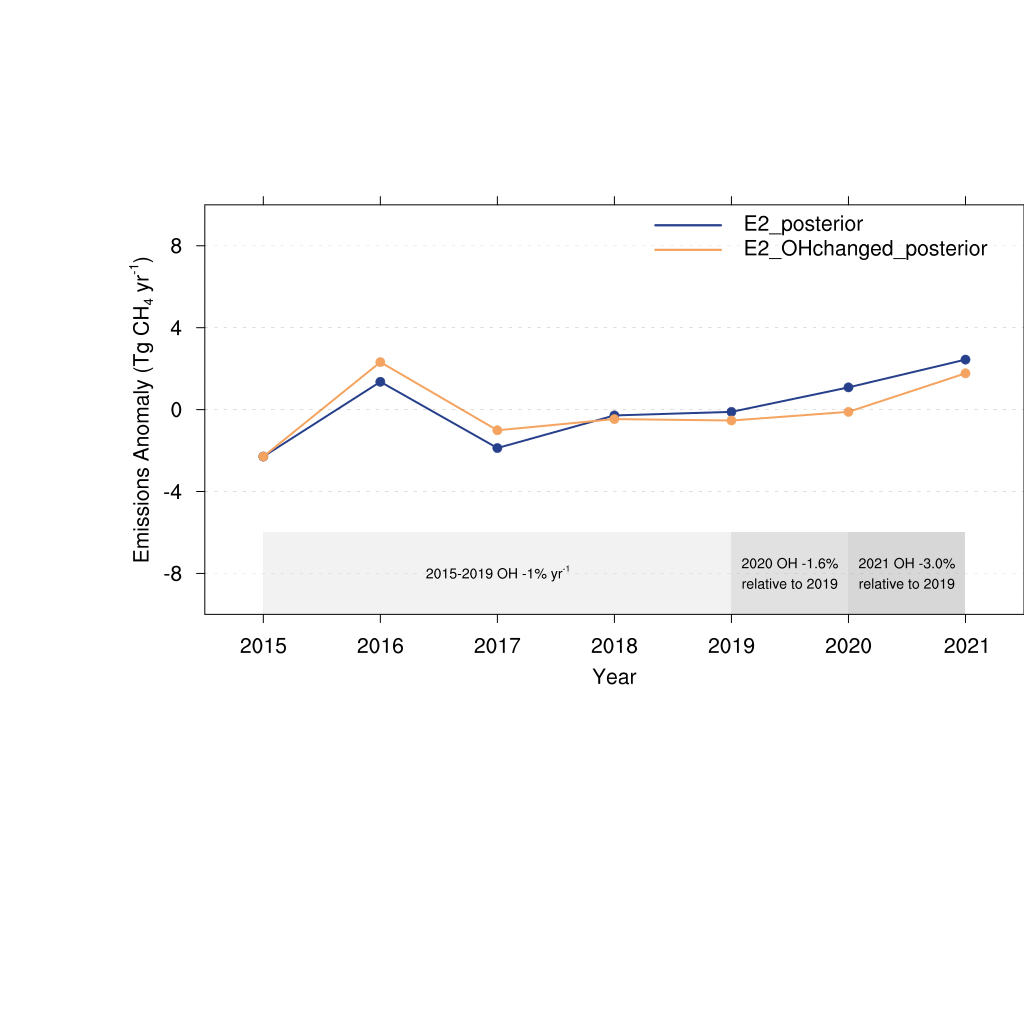


**Fig. S23**. Posterior methane emission anomaly in China from E2 experiment and E2_OHchanged experiment during 2015-2021.


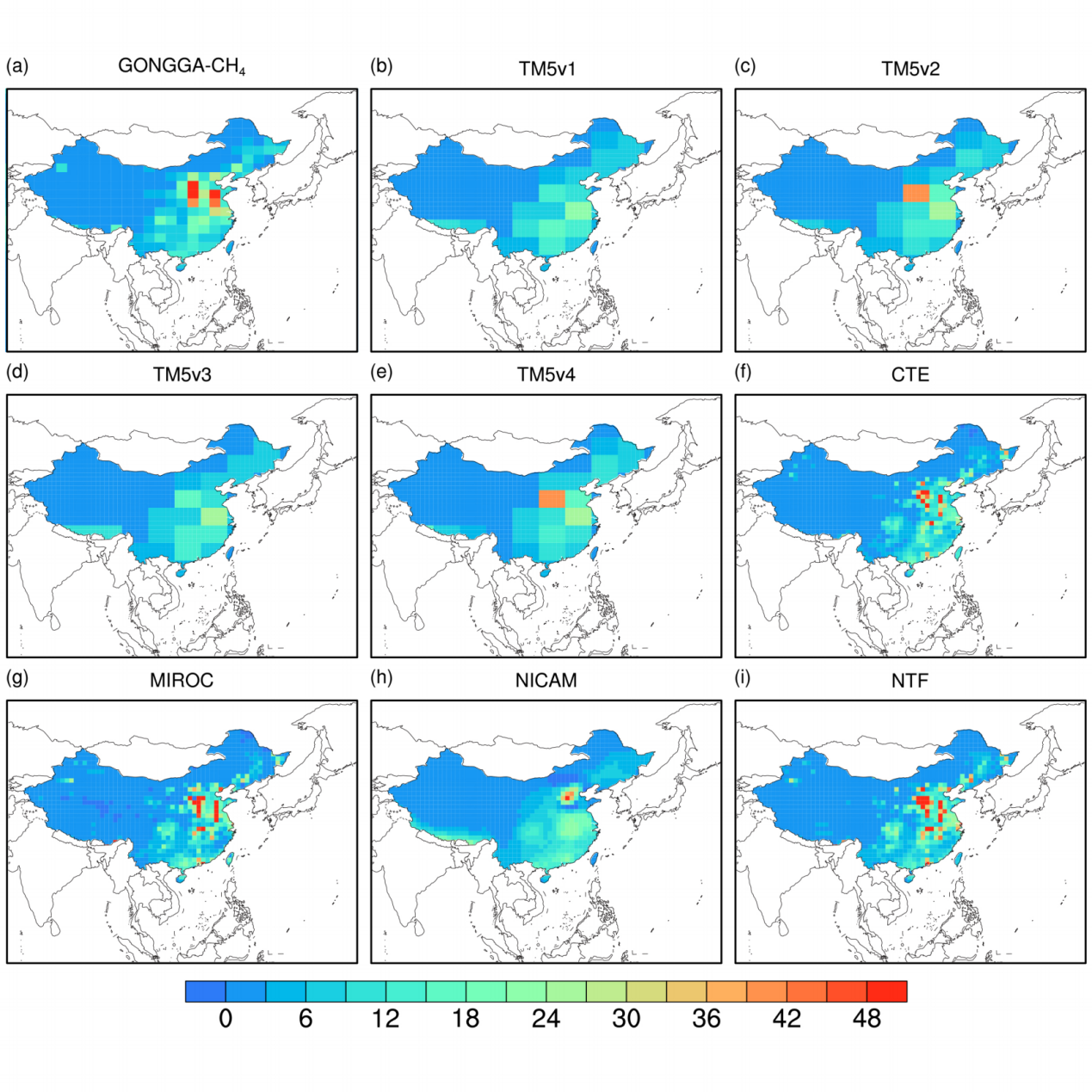


**Fig. S24**. Spatial distributions of the annual mean methane emissions for different inversions.

**Supplementary Table**

**Table S1.** List of inversion setups.

| Inversion  Experiment | Prior  (Anthropogenic) | Prior  (Wetlands) | Prior  (biomass burning) |
| --- | --- | --- | --- |
| E1 | EDGAR v6.0 | JPL_WetCharts | GFED4 |
| E2 | EDGAR v7.0 | JPL_WetCharts | GFED4 |
| E3 | CTE posterior | CTE posterior | CTE posterior |

**Table S2**. The methane emissions during 2011-2021 in different regions of China.

| Emissions  (Tg CH_4_ yr^-1^) | Northwest | Northeast | North | South | Southwest | Tibetan Plateau |
| --- | --- | --- | --- | --- | --- | --- |
| Agriculture and Wastes | 2.24±0.41 | 2.29±0.34 | 7.09±1.13 | 14.78±2.50 | 5.16±0.56 | 1.04±0.20 |
| Fossil Fuels | 2.51±1.01 | 1.98±0.23 | 13.59±5.36 | 2.94±1.00 | 0.76±0.24 | 0.09±0.01 |
| Wetlands | 0.32±0.04 | 0.50±0.10 | 0.28±0.19 | 2.16±0.79 | 0.29±0.08 | 0.40±0.13 |
| Biomass and Biofuel Burning | 0.11±0.09 | 0.12±0.07 | 0.41±0.37 | 0.67±0.57 | 0.35±0.30 | 0.03±0.02 |

**References:**

1. Butz A, Guerlet S, Hasekamp O *et al.* Toward accurate CO2 and CH4 observations from GOSAT. *Geophysical Research Letters*. 2011; **38**. doi: 10.1029/2011gl047888

2. Kuze A, Suto H, Shiomi K *et al.* Update on GOSAT TANSO-FTS performance, operations, and data products after more than 6 years in space. *Atmospheric Measurement Techniques*. 2016; **9**(6): 2445-2461. doi: 10.5194/amt-9-2445-2016

3. Parker RJ, Webb A, Boesch H *et al.* A decade of GOSAT Proxy satellite CH4 observations. *Earth System Science Data*. 2020; **12**(4): 3383-3412. doi: 10.5194/essd-12-3383-2020

4. Wunch D, Toon GC, Blavier J-FL *et al.* The Total Carbon Column Observing Network. *Philosophical Transactions of the Royal Society a-Mathematical Physical and Engineering Sciences*. 2011; **369**(1943): 2087-2112. doi: 10.1098/rsta.2010.0240

5. Masarie KA, Peters W, Jacobson AR *et al.* ObsPack: a framework for the preparation, delivery, and attribution of atmospheric greenhouse gas measurements. *Earth System Science Data*. 2014; **6**(2): 375-384. doi: 10.5194/essd-6-375-2014

6. Yan XY, Cai ZC, Ohara T *et al.* Methane emission from rice fields in mainland China: Amount and seasonal and spatial distribution. *Journal of Geophysical Research-Atmospheres*. 2003; **108**(D16). doi: 10.1029/2002jd003182

7. Zhang Y, Fang S, Chen J *et al.* Observed changes in China's methane emissions linked to policy drivers. *Proceedings of the National Academy of Sciences of the United States of America*. 2022; **119**(41): e2202742119-e2202742119. doi: 10.1073/pnas.2202742119

8. Montzka SA, Krol M, Dlugokencky E *et al.* Small Interannual Variability of Global Atmospheric Hydroxyl. *Science*. 2011; **331**(6013): 67-69. doi: 10.1126/science.1197640

9. Rigby M, Montzka SA, Prinn RG *et al.* Role of atmospheric oxidation in recent methane growth. *Proceedings of the National Academy of Sciences of the United States of America*. 2017; **114**(21): 5373-5377. doi: 10.1073/pnas.1616426114

10. Turner AJ, Frankenbergb C, Wennberg PO *et al.* Ambiguity in the causes for decadal trends in atmospheric methane and hydroxyl. *Proceedings of the National Academy of Sciences of the United States of America*. 2017; **114**(21): 5367-5372. doi: 10.1073/pnas.1616020114

11. Peng S, Lin X, Thompson RL *et al.* Wetland emission and atmospheric sink changes explain methane growth in 2020. *Nature*. 2022; **612**(7940): 477-482. doi: 10.1038/s41586-022-05447-w

12. Lin X, Peng S, Ciais P *et al.* Recent methane surges reveal heightened emissions from tropical inundated areas. *Preprint*. 2023. doi: 10.31223/X5ZH4S

13. Miller SM, Michalak AM, Detmers RG *et al.* China's coal mine methane regulations have not curbed growing emissions. *Nat Commun*. 2019; **10**(1): 303. doi: 10.1038/s41467-018-07891-7

14. Sheng J, Tunnicliffe R, Ganesan AL *et al.* Sustained methane emissions from China after 2012 despite declining coal production and rice-cultivated area. *Environmental Research Letters*. 2021; **16**(10). doi: 10.1088/1748-9326/ac24d1

15. Zhang Y, Jacob DJ, Lu X *et al.* Attribution of the accelerating increase in atmospheric methane during 2010-2018 by inverse analysis of GOSAT observations. *Atmospheric Chemistry and Physics*. 2021; **21**(5): 3643-3666. doi: 10.5194/acp-21-3643-2021

16. Stavert AR, Saunois M, Canadell JG *et al.* Regional trends and drivers of the global methane budget. *Global Change Biology*. 2022; **28**(1): 182-200. doi: 10.1111/gcb.15901
